# Phytochemical Analysis and Habitat Suitability Mapping of *Cardiocrinum cordatum* (Thunb.) Makino Collected at Chiburijima, Oki Islands, Japan

**DOI:** 10.3390/molecules27238126

**Published:** 2022-11-22

**Authors:** Fuzuki Momotomi, Aedla Raju, Dongxing Wang, Doaa H. M. Alsaadi, Takashi Watanabe

**Affiliations:** 1 Department of Medicinal Plant, Graduate School of Pharmaceutical Sciences, Kumamoto University, 5-1 Oe-Honmachi, Chuo-ku, Kumamoto 862-0973, Japan; 2 Global Center for Natural Resources Sciences, Kumamoto University, No. 5-1, Oe Honmachi, Chuo-ku, Kumamoto 862-0973, Japan; 3 BVRIT HYDERABAD College of Engineering for Women, Nizampet Rd, Hyderabad 500090, Telangana, India

**Keywords:** *Cardiocrinum cordatum*, ACE inhibition, antioxidant activity, soil moisture content, geographic information system, habitat suitability map, MaxEnt

## Abstract

*Cardiocrinum cordatum*, known as ubayuri in Japan, has antihypertensive properties and has been shown to inhibit angiotensin-converting enzyme (ACE), which contributes to the production of angiotensin II, a hypotensive substance in the renin–angiotensin system. *C. cordatum* has been the subject of various studies as a useful plant and is applied as a functional food. Due to the limited distribution, loss of natural habitat by frequent natural disasters, and environmental conditions, the chemical content and biological activity of *C. cordatum* have been drastically affected. Obtaining a stable supply of *Cardiocrinu cordatum* material with high biological activity is still a challenge. Understanding the native habitat environment and suitable cultivation sites could help in solving this issue. Therefore, in the current study we investigated the effect of environmental parameters on the hypertensive and antioxidant activities of *C. cordatum* collected at Chiburijima, Oki Islands, Shimane Prefecture, Japan. We also predicted the habitat suitability of *C. cordatum* using a geographic information system (GIS) and MaxEnt model with various conditioning factors, including the topographic, soil, environmental, and climatic factors of the study area. A total of 37 individual plant samples along with soil data were collected for this study. In vitro assays of ACE inhibitory and antioxidant activity were conducted on the collected samples. The results show that plants at 14 out of 37 sites had very strong ACE inhibitory activity (IC_50_ < 1 mg mL^−1^). However, the collected plants showed no signs of strong antioxidant activity. Statistical analysis using analysis of variance (ANOVA) showed that BIO05 (F value = 2.93, *p* < 0.05), nitrate–nitrogen (F value = 2.46, *p* < 0.05), and silt (F value = 3.443, *p* < 0.05) significantly affected ACE inhibitory activity. On the other hand, organic carbon content (F value = 10.986, *p* < 0.01) was found to significantly affect antioxidant activity. The final habitat suitability map shows 3.3% very high and 6.8% high suitability regions, and samples with ACE inhibition activity were located within these regions. It is recommended further investigations and studies are conducted on *C. cordatum* in these locations. The prediction suitability model showed accuracy with AUC-ROC of 96.7% for the study area.

## 1. Introduction

*Cardiocrinum cordatum* (Thunb.) Makino, belonging to Liliaceae, is a perennial herb that grows in moderate deciduous forests. In Japan, it is distributed in Honshu, Shikoku, and Kyushu, where it is known by the name ubayuri [1]. *C. cordatum* is also native to Northeast Asia and the Russian Far East [2,3]. *C. cordatum* is used as a medicinal plant in Japan and China [4]. The scales are especially edible, and the starch extracted from the scales is used as medicine and food. The fiber produced from the scales is also used as an ingredient in several traditional sweets [5,6,7]. In addition, the scales are used as an ingredient in traditional Japanese foods such as tempura and chawanmushi (steamed egg custard). The leaves and petioles are also used as functional food [8]. In addition, the seeds of *C. cordatum* have antipyretic properties, which make them useful as a medicine to cure colds [9].

According to Okazaki et al., 70% EtOH extract of the leaf blade or stem of *C. cordatum* has high angiotensin-converting enzyme (ACE) inhibitory activity. In addition, experiments with *C. cordatum* leaf extract administered to SHR/Izm (spontaneously hypertensive) rats showed a concentration-dependent antihypertensive effect [10]. ACE is an enzyme that plays a central role in the renin–angiotensin system, known as the pressure-raising system, and the kallikrein–kinin system, known as the pressure-lowering system, and is responsible for blood pressure regulation. *C. cordatum* inhibits ACE, thereby reducing its concentration in the renin–angiotensin system and inhibiting the degradation of bradykinin in the kallikrein–kinin system, resulting in synergistic hypotensive effects. In addition, ACE has also been reported to be associated with various diseases, including diabetes, cerebrovascular and cardiovascular diseases [11], and COVID-19 [12]. Although there are no reports of antioxidant properties of *C. cordatum*, plants from the same family have been found to have antioxidant properties [13].

Agricultural activities and natural disasters have reduced the population of *C. cordatum* and affected the genetic diversity of the plant [9]. Therefore, its economic value has increased due to overexploitation and loss of natural habitats. Appropriate environmental conditions (soil, water, vegetation) efficiently supply nutrients to the plant, leading to increased plant richness, component content, and pharmacological functions [14,15]. Atmospheric temperature normalizes the rate of plant growth and development [16], while water stress causes leaf shrinkage, shortened tops, disrupted plant growth, delayed flowering and fruiting, reduced seed number and size, and death. Soil properties such as pH, electrical conductivity (EC), and water content play a major role in plant growth [17]. Soil pH has been shown to directly affect the protoplasm of plant root cells, as well as the microbial activity of nutrients, nutrient content, and toxicity [16,17]. Essential nutrients such as calcium, magnesium, potassium, phosphorus, and molybdenum are washed out and deficient in acidic soils (pH < 6) [18]. Nitrogen concentrations are also low, especially in acidic soils, and nitrogen is available in the form of ammonium, while nutrients such as zinc, iron, copper, potassium, and manganese have lower concentrations in basic soils with pH > 8. In other words, not all plant species are adapted to the same level of pH for survival [18,19]. Soil moisture is a major resource for plant growth, productivity, and nutrient richness [20,21,22] and can also affect species diversity [23]. This demands more efficient measures for the surveying, protection, cultivation, consumption, and harvesting of these species, including their geographic distribution, biological activity, relationship with environmental parameters, and genetic analysis.

A geographic information system (GIS) can provide efficient statistical and spatial modeling results by integrating various environmental datasets to develop habitat suitability maps (HSMs) for species conservation. Habitat suitability models define the relationships between species and their environment based on an understanding of their geographic distribution [24]. Species distribution models (SDMs) are of great value in ecological studies because of the established relationships between species frequency and various biophysical and ecological variables [25,26,27,28]. Generalized linear models (GLMs) and generalized additive models (GAMs) [25,29], general rule set generation algorithms (GARPs) [24,30], maximum entropy (MaxEnt) [24,31,32], bioclimatic envelopes (BioClim) and DOMAIN, support vector machine (SVM) models [33,34], and other species distribution models with different concepts have been used for habitat suitability maps.

Previous studies have shown that polyphenols and flavonoids in foods such as tea contribute to ACE inhibitory and antioxidant activities [35,36,37]. Therefore, the aim of this study was to identify the parameters that affect the biological activities of *C. cordatum* and its habitat suitability. In this paper, an integrated analysis of *C. cordatum* collected in Chiburijima was performed. The plant samples along with soil data, including soil moisture content, soil bearing capacity, and pH, were collected. Soil samples were also collected for chemical analysis. Finally, using the collected geographic factors and various influence factors of the plants, a habitat suitability map was created to predict the distribution of the plants in the study area.

## 2. Results and Discussion

### 2.1. Soil Analysis

Table 1 shows the results of soil macronutrients from *C. cordatum* collection sites. The major macronutrients in the soil were examined for statistical correlation with ACE inhibitory and radical scavenging activities. The highest activity of ACE inhibition was found in sample Ch-2, which had high levels of potassium and magnesium and a moderate level of calcium when compared to sample Ch-27, which had the lowest ACE inhibitory activity and a difference in soil macronutrients by nearly double or more. Similar results obtained from other studies suggested a close correlation between soil parameters and the biological activities of plants [38,39].

### 2.2. ACE Inhibition, DPPH Activity, and Total Phenolic and Flavonoid Content

The results in Table 2 show that most of the collected *C. cordatum* plants had high-to-moderate ACE inhibitory activity. Ch-16, Ch-21, Ch-24, and Ch-27 showed weak ACE inhibitory activity. On the other hand, the level of radical scavenging activity was very low at all collection sites. There was no correlation between ACE inhibitory activity and antioxidant activity. Although previous studies have reported that polyphenols and flavonoids in tea and other foods contribute to ACE inhibitory and antioxidant activity [35,36,37], the present results suggest that polyphenols are unlikely to contribute to such activity. Table 2 shows very low values for total phenolic and flavonoid content, which explains the weak antioxidant activity of the collected samples. Previous studies have not reported on components found in *C. cordatum* extracts that contribute to ACE inhibitory activity. However, many other vegetables, including onions and chives, which are members of the same lily family as *C. cordatum*, as well as mushrooms, are rich in nicotianamine, which has attracted attention as a substance that may contribute to ACE inhibitory activity [40]. Peptides, which are available on the market, are used as ingredients in healthy foods for their blood pressure-lowering properties and are thought to have the potential to contribute to ACE inhibitory activity. Conditional factors of the plant collection sites for statistical analysis are shown in Supplementary Table S1. ANOVA of ACE inhibitory and antioxidant activity of *C. cordatum* with various conditioning factors of the study area are shown in Supplementary Table S2.

Since Chiburijima is surrounded by the sea and affected by seasonal winds, it is quite possible that experimental results will be affected by climate, soil, and other factors. Therefore, ANOVA was conducted to see if a correlation could be found between ACE inhibitory activity and antioxidant activity and environmental parameters (Table 3). As a result, three factors were found to significantly affect ACE inhibitory activity: maximum temperature of the warmest month (BIO05), silt, and nitrate–nitrogen. Meanwhile, organic carbon content was found to significantly affect antioxidant activity. The results of soil composition analysis showed that the content of phosphorus, potassium, and calcium varied from site to site. These results suggest that mainly soil factors contribute to ACE inhibitory and antioxidant activity.

### 2.3. Habitat Suitability Map (HSM)

**Figure 1:**
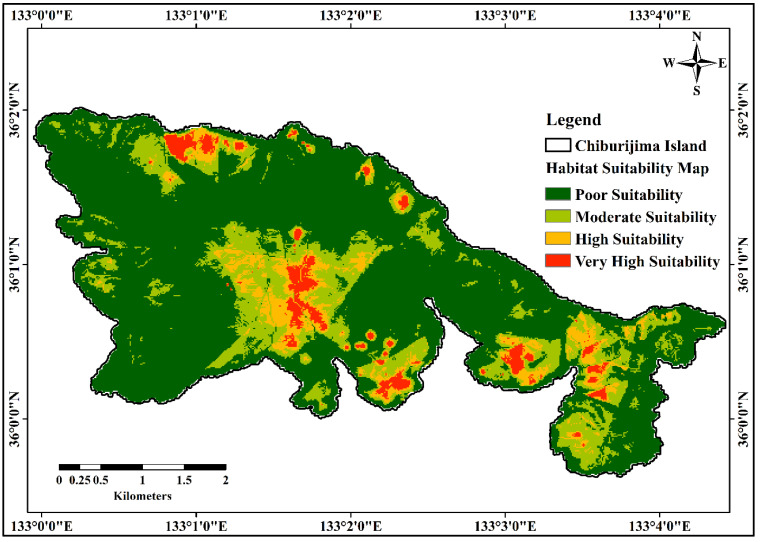
Habitat suitability map of predicted *C. cordatum* using MaxEnt model.

Habitat suitability was predicted using the maximum entropy (MaxEnt) model with 324 occurrence records of *C. cordatum* and 32 effective conditioning factors of the study area. The final map was classified into four suitability classes (low, moderate, high, and very high) as per the natural break classification technique in ArcGIS, as shown in Figure 1. The statistical correlation between conditioning factors and species occurrence provided an appropriate spatial distribution and habitat suitability of *C. cordatum* in the study area.

The conditioning factors have relative variable importance to the distribution of *C. cordatum* species within their habitat. In general, climatic factors such as temperature and precipitation have a destructive effect on environmental layers related to species distribution and habitat suitability prediction using the MaxEnt model. The occurrence and distribution pattern of *C. cordatum* species in natural habitats depend on topographical, soil, climatic, and environmental factors. The effect of relative variable importance on the conditioning factors was assessed using the jackknife variance estimation process for the area under the curve (AUC).

In the study area, conditioning factors, such as slope, aspect, hillshade, soil pH, soil bulk density, soil clay, precipitation of the driest month (BIO14), minimum temperature of the coldest month (BIO06), and maximum temperature of the warmest month (BIO05), had little importance in the effect on habitat suitability. Distance to road, distance to urban area, distance to stream, soil OCC, cation exchange capacity (CEC), soil moisture sensor (SM150T output), and elevation are important in predicting suitable areas of *C. cordatum* growing in the study area. Annual mean temperature (BIO01), temperature, precipitation, soil electrical conductivity (EC), and digital elevation model (DEM) have a strong correlation with species occurrence in habitat prediction.

The study area is located at 22–350 m above sea level so, the DEM and elevation show the most significant effect on habitat suitability. However, the topographical factors extracted from DEM, such as slope, aspect, curvature, plan and profile curvature, TWI and hillside, have no strong effect on habitat suitability prediction for *C. cordatum* species. All soil parameters considerably influence habitat suitability. During data collection, it was observed that most of the soil had lower percentages of sand, silt, and clay; therefore, it also did not show any important effect on habitat suitability. Among the soil factors, EC, soil bearing capacity, OCD, and pH in H_2_O have an important effect on suitability prediction for *C. cordatum*. After that, soil moisture (SM150T output), CEC, soil bulk density, organic carbon content (OCC), and pH influence suitability estimation.

The plant samples were collected at a distance of 0.3–40 m from the road, 0.2–806 m from the urban area, and 6–632 m distance from the stream, based on the accessibility and maturity of plants. The distance of the plant location has a significant influence on habitat prediction.

**Figure 2:**
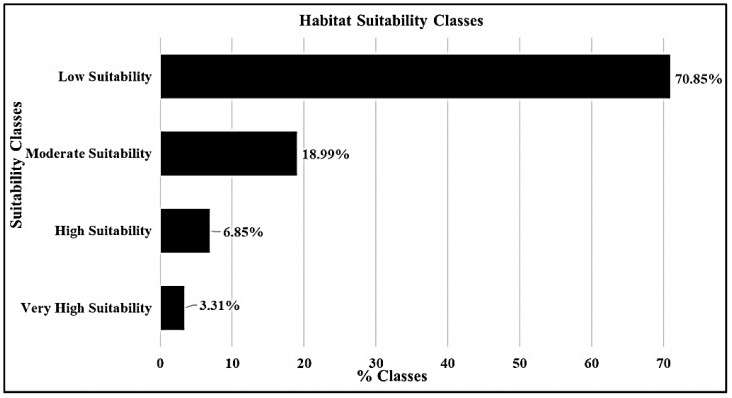
Percentages of habitat suitability classes.

For the study area, according to the habitat suitability map, very high and high suitability zones covered 3.3 and 6.8% of the total area, respectively. The low suitability zone occupied a large proportion, 70.8%, and the moderately suitability zone occupied 18.9% of the total study area, as shown in Figure 2.

Samples with very strong ACE inhibition activity were located in the very high and high suitability zones. These locations are highly recommended for further collection, cultivation, and investigation of *C. cordatum*. This could help to advance drug discovery and functional food development research on *C. cordatum*. Most of the samples with weak ACE inhibition activity were located in the moderate and poor suitability zones. Samples such as 27, 21, and 28 had low ACE inhibition activity, but were located in the high suitability zone. From the suitability analysis and field observations, it was concluded that these samples were collected in areas with complete shade and high-moisture soil, which might be the reason for the contrast with respect to plant suitability in the study area.

### 2.4. Importance of Effective Factors

**Figure 3:**
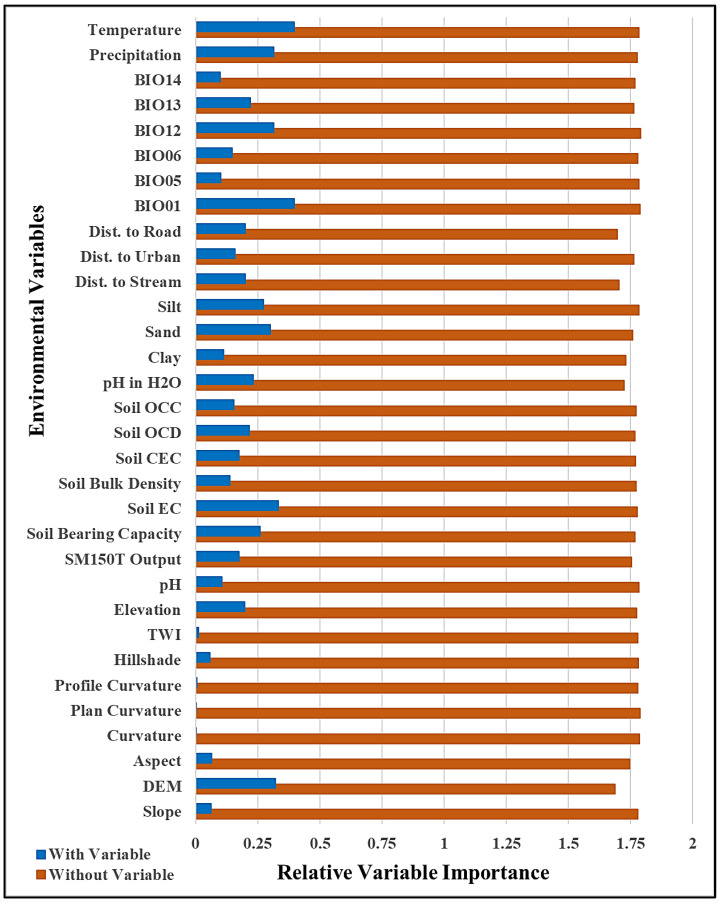
Analysis of relative importance of effective environmental variables.

Environmental factors play a significant role in the geographic distribution and density of plant species. It is necessary to evaluate the relative variable importance of different factors on the habitat suitability of *C. cordatum*. In this study, the jackknife variance estimation method for the area under the curve (AUC) was used, and the analysis results are shown in Figure 3. The results of variable importance show that temperature, annual mean temperature (BIO01), soil electrical conductivity (EC), and digital elevation model (DEM) had the highest importance for HSM. Precipitation, annual precipitation (BIO12), silt, sand, and soil bearing capacity had moderate importance in developing HSM. In contrast, the AUC did not show significance in HSM modeling. It is always necessary to identify and delineate various environmental variables that affect plant habitats in order to develop habitat suitability maps of several plant resources in every location.

### 2.5. Validation of MaxEnt Model

**Figure 4:**
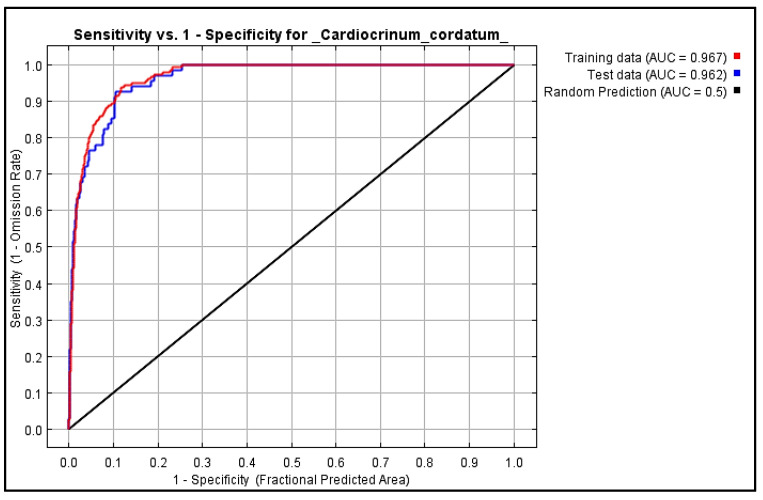
Receiver operator characteristic (ROC) curve for habitat suitability map of *C. cordatum* produced by MaxEnt model.

The predicted habitat suitability map of *C. cordatum* was validated using the receiver operator characteristic (ROC) curve. The MaxEnt spatial distribution model was evaluated by AUC value, as illustrated in Figure 4. The results show that the model exhibited outstanding performance, with AUC values of 96.7% (training data) and 96.2% (test data). The model exhibited logical, adequate, and respectable accuracy output for calculating the habitat suitability of *C. cordatum*.

## 3. Materials and Methods

### 3.1. Study Area

**Figure 5:**
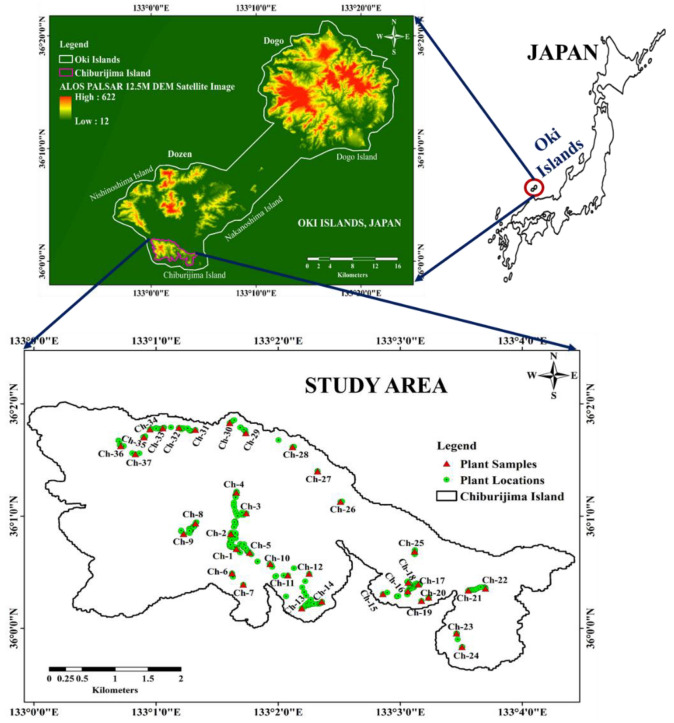
Plant locations and sample map of study area, which comprises an area of 11.4 km^2^ of Chiburijima. Map was prepared using ALOS PALSAR DEM satellite image and ArcGIS version 10.5.1 (Licensed).

The Oki Islands are located in the Sea of Japan in the southern part of Oki District, Shimane Prefecture, Japan. These islands are situated between the island arc of Japan and the Eurasian continent, within the marginal sea represented by the Sea of Japan. The islands are separated into Dozen and Dogo Islands, which lie within a 40 km north–south and east–west perimeter. Nishinoshima, Nakanoshima, and Chiburi Islands are together known as the Dozen Islands, which are close to the mainland. Chiburi Island (Chiburijima), an area of 11.4 km^2^, which lies between 36°0′0″–36°2′0″ N latitude and 133°0′0″–133°5′0″ E longitude, was considered as the study area (as shown by ALOS PALSAR DEM satellite image and ArcGIS version 10.5.1 (ESRI Japan Corporation, Tokyo, Japan)) (Figure 5). It is one of the smallest islands of Dozen, with a maximum elevation of 325 m above sea level. The temperature ranges from 34.9 to −2.9 °C, and the average annual rainfall is about 1662 mm, according to the Meteorological Agency of Japan [41]. Chiburijima features complex and fascinating landforms that were developed by erosion and weathering of volcanic rocks. This island has a unique ecosystem, and there are deep connections among the land, plants, and animals, which represent the culture and people’s lifestyles.

### 3.2. Plant Materials and Data Collection

The plant species were identified during field investigations in Chiburijima, and 324 positions were recorded using a global positioning system (GPS) device (Garmin eTrex 30×, Olathe, KS, USA). Thirty-seven plant samples were collected for the ACE inhibitory and antioxidant activity experiment. Most of the species were located next to traffic-free roads and in the shade with high-moisture soil. The strain of the collected samples has the green color axis. Some of the species were recorded under sunlight, with light to dark yellow colored leaves. The species were not severely exposed to high concentrations of pollutants, and the atmosphere on the island is calm and pollution-free. Photographs of the *C. cordatum* sample collection are shown in Supplementary Figure S1.

### 3.3. Soil Analysis

During the field survey, soil pH and bearing capacity and soil moisture levels were measured using a conventional pH meter, a Yamanaka-type soil hardness tester (Fujiwara Seisakusho, Ltd., Tokyo, Japan), and a soil moisture sensor kit (SM150T, Delta-T Devices, Cambridge, UK). The recordings of all 37 plant locations are listed in Table 4.

The collected soil samples were analyzed using a soil analyzer (EW-THA1J, Air Water Biodesign Co., Ltd., Kobe, Hyogo-Pref., Japan), and the resulting macronutrient values are shown in Table 1.

### 3.4. Preparation of Plant Extract

Leaves of *C. cordatum* were dried at 50 °C for 3 days, and each sample was blended to form a powder for the extraction experiment. Approximately 1 g of plant powder was mixed with 20 mL of 70% ethanol and sonicated at 50 °C for 60 min. These suspensions were then mixed at 25 °C for another 24 h with shaking at 85 rpm. After filtration, the filtrate was concentrated and put through a decompression dryer to dry under vacuum pressure overnight. The dried extract was dissolved in water and stored at −30 °C for ACE inhibition and antioxidant activity studies.

### 3.5. Chemicals

Sodium tetraborate decahydrate and boric acid were purchased from Nacalai Tesque (Kyoto, Japan). Angiotensin-converting enzyme (ACE) and Folin–Ciocalteu phenol reagent were obtained from Sigma-Aldrich (Tokyo, Japan), and histidyl–leucine (HL) from Peptide Laboratory (Osaka, Japan). Captopril, hippuryl–histidyl–leucine (HHL), O-phthalaldehyde, 2-morpholinoethanesulphonic acid (MES), Trolox, and dimethyl sulfoxide (DMSO) were obtained from Fujifilm Wako Pure Chemical Industries (Osaka, Japan), and 1,1-diphenyl-2-picrylhydrazyl (DPPH) was obtained from Rakuto Kasei (Tokyo, Japan). Gallic acid was bought from Tokyo Chemical Industry (Tokyo, Japan). Sodium carbonate (anhydrous) and aluminum chloride (III) (anhydrous) were acquired from Kishida Chemical Co., Ltd. (Osaka, Japan). Quercetin was purchased from Funakoshi Co., Ltd. (Tokyo, Japan).

### 3.6. Instrumentation

A SpectraMax iD3 (Molecular Devices, Tokyo, Japan) was used to measure absorbance in the antioxidant activity test, and a Cytation 5 Cell Imaging Multi-Mode Reader (BioTek, Tokyo, Japan) was used to measure fluorescence in the ACE inhibitory activity test.

### 3.7. ACE Inhibition

The evaluation of ACE inhibitory activity was performed using a slightly modified version of the method of Cushman and Cheung [42]. Briefly, a dilution series of sample and captopril were prepared: ACE solution (144 µL, 4 mU/mL), HHL solution (36 µL, 20 mM), O-phthalaldehyde (10 µL, 20 mg/mL), and sample or captopril were added to prepare reaction solutions in 96-well plates. Calibration solutions were then prepared by adding borate buffer (60 µL, pH 8.3), HL solution (15 µL, 0.1 mM), NaOH (30 µL, 1N), sample or captopril (30 µL), and *O*-phthalaldehyde (10 µL). All reagents were freshly prepared. The reaction of HHL with ACE produces hippuric acid (HA) and HL, and HL fluoresces under alkaline conditions due to O-phthalaldehyde. The plates were then incubated in the dark at room temperature for 12 min. Then, HCl (10 µL, 1 N) was added to the calibration and reaction solutions in the plate to stop the reaction. Finally, the fluorescence intensity was measured using the plate reader (excitation wavelength: 355 nm; emission wavelength: 460 nm).

The ACE inhibition rate was calculated by the following Equation (1):

(1) ACE inhibition rate (%)=(1−B/A)×100

where A is the HL concentration in the absence of sample or captopril, and B is the HL concentration in the presence of sample or captopril.

### 3.8. Radical Scavenging Activity by DPPH Method

Antioxidant activity was measured by a method slightly modified from the methods of Oki et al. and Maeda et al. [43,44]. All reagents were freshly prepared. Briefly, a dilution series of samples and Trolox were prepared. In 96-well plates, 20 µL of sample solution, 50% EtOH solution (180 µL per well), MES buffer (50 µL per well, 200 mM, pH 6.0), and DPPH solution (50 µL per well, 800 µM) were placed in each well. Trolox (20 µL per well, 1.6 mM) was added to 50% EtOH solution (180 µL per well), MES buffer (50 µL per well, 200 mM), and DPPH solution (50 µL per well, 800 µM). A blank solution was also prepared by adding 50% EtOH solution (150 µL per well) and MES buffer (50 µL per well, 200 mM). The reaction of DPPH with the sample or Trolox induced radical-scavenging activity in the DPPH molecules, changing the color from purple to yellow. Antioxidant activity can be measured by the intensity of absorbance. The plates were then incubated in the dark at room temperature for 20 min. Finally, absorbance intensity was measured using the plate reader (absorbance wavelength: 520 nm).

A calibration curve was generated from the Trolox concentration and absorbance. For the sample solutions, absorbance values for each well were created from the sample concentration and absorbance. Additionally, each absorbance value was subtracted from the blank value. Antioxidant activity (%) was calculated using the following Equation (2):

(2) DPPH radical scavenging rate (%)=(1−B/A)×100

where A is the control absorbance of DPPH radicals without the sample, and B is the absorbance after reacting with the sample. The values of A and B were obtained by subtracting the value of the blank (influenced by the plate and sample solution).

### 3.9. Total Phenolic Content

Total polyphenol content was determined by the Folin–Ciocalteu method, a modification of the method reported by Riitta Julkunen-Tiitto et al. [45]. Briefly, 70% EtOH extract (20 µL per well) and 10% Folin–Ciocalteu phenol reagent (100 µL per well) were added to a 96-well plate. After 5 min, 2.5% sodium carbonate solution (80 μL per well) was added and allowed to stand for 1 h in the dark at room temperature. The absorbance was measured using the plate reader (absorbance wavelength: 755 nm). A calibration curve was prepared using gallic acid. The total polyphenol content was determined as the equivalent of gallic acid (mg g^−1^ extract) based on the gallic acid calibration curve (y = 0.0217x + 0.0561, R^2^ = 0.9673).

### 3.10. Total Flavonoid Content

The flavonoid assay was performed by using a modified version of the method reported by Pękal et al. [46]. In a 96-well plate, 70% EtOH extract solution (25 μL per well) and 80% EtOH (75 μL per well) were added. Then, 10% aluminum chloride (III), 10% aluminum chloride (III) MeOH solution (5 μL per well), and pure water (145 μL per well) were added. The mixture was covered with aluminum foil and allowed to stand at room temperature for 30 min. The absorbance was measured using the plate reader (absorbance wavelength: 420 nm). A calibration curve was prepared using quercetin. The total flavonoid content was determined as the equivalent of quercetin (mg g^−1^ extract) based on the quercetin calibration curve (y = 0.0576x + 0.0099, R^2^ = 0.9996).

### 3.11. Dataset Preparation for Habitat Suitability Mapping

Various conditioning factors, such as topographic, climatic, soil, and environmental factors, are necessary to predict the appropriate habitat suitability of *C. cordatum* using an efficient spatial distribution model. In total, 32 essential conditioning factors were prepared for predicting the habitat suitability map using MaxEnt, a machine learning-based spatial modeling approach. The boundary map of Chiburijima was prepared with a 1:50,000 scale toposheet from the Geological Survey of Japan (GSJ) https://www.gsj.jp/en/database/index.html (accessed on 19 August 2020), using Google Earth Pro and GIS software (ArcGIS version 10.5.1). Geographical factors, includin19g slope, aspect, curvature, plan curvature, profile curvature, and hillshade, were extracted from the Advanced Land Observing Satellite–Phased Array Type l-Band Synthetic Aperture Radar–Digital Elevation Model (ALOS PALSAR DEM) satellite image with 12.5 m × 12.5 m spatial resolution https://vertex.daac.asf.alaska.edu (accessed on 20 August 2020) [47]. The slope map was extracted, and the surface direction with the degree of slope was represented through aspect. The aspect map was prepared with five classes: flat, north, east, south, and west. A curve with the rate of change of slope and extended plant curvature and profile curvature maps was also prepared. The extracted hillshade map represents topographical forms of highlands of the study area. A topographic wetness index (TWI) map was also prepared, using the slope and the upstream cover area, to estimate where water will accumulate in areas with varying elevation.

Soil factors, including pH in H_2_O, bulk density, CEC, clay content, sand content, silt content, OCD, and OCC, were downloaded from SoilGrids250m 2.0 http://soilgrids.org (accessed on 20 August 2020) at a depth of 0.15 m. Six bioclimatic variables (BIO01, BIO05, BIO06, BIO12, BIO13, and BIO14) out of 19 variables [48] related to temperature and precipitation were also considered, and these were downloaded from the WorldClim website https://www.worldclim.org/ (accessed on 26 August 2020). In addition, the climatic factors of precipitation and temperature were collected from the Japan Meteorological Agency https://www.jma.go.jp/ (accessed on 28 August 2020). Maps of species’ distance from roads, streams, and urban areas were prepared using topographical maps (1:250,000). The elevation map was prepared by interpolating altitude (m) values of each plant location collected using a GPS device. Thematic maps of soil factors such as soil moisture content, soil bearing capacity, and pH were also prepared from the field survey data. These maps were interpolated by applying the inverse distance weighted (IDW) spatial analysis technique in ArcGIS. All maps were prepared in raster form (.tif) and then converted into ASCII (.ASC) format with the same spatial resolution (12.5 m × 12.5 m) for further analysis. Thematic maps of various conditioning factors with plant locations used for predicting the habitat suitability of *C. cordatum* are shown in Supplementary Figure S2. The list of conditioning factors is shown in Table 5.

### 3.12. Maximum Entropy (MaxENT) Model and Validation

MaxEnt is an efficient machine learning-based spatial distribution modeling approach for determining geographically distributed species in their natural habitat [28]. It is used to predict the spatial distribution and habitat suitability of species based on presence-only occurrence data [28,49]. MaxEnt is used to estimate the spatial distribution of target species by defining the probability distribution of species occurrence and various conditioning factors related to climate, topography, environment, and soil. The MaxEnt model is a direct way to create habitat suitability with great use of data entropy, which is used to select data and obtain unexpected results of the model [50]. MaxEnt is efficient for small-size species presence-only data and also delivers good results even for incomplete data [51,52]. In the MaxEnt model, both categorical and continuous formats of environmental layers and species data are constant, correct, and reliable, even if the numbers are small. It is capable of predicting the habitat suitability of targeted species with appropriate outcomes, which is advantageous for mapping, future cultivation, and conservation of species in their natural habitat [52,53,54].

In this study, MaxEnt software version 3.4.1 was downloaded from the website of the Center for Biodiversity and Conservation at the American Museum of Natural History (AMNH) https://biodiversityinformatics.amnh.org/open_source/maxent/ (accessed on 23 August 2020), and was employed to predict the habitat suitability of *C. cordatum* with 324 species-occurrence records and 32 conditioning factors. For modeling, 70% of the identified and georeferenced samples (226 plants) were randomly selected as training data and the remaining 30% (98 plants) as testing data to validate the MaxEnt model. The relative importance of conditioning factors was assessed using the jackknife test [55]. The model-generated output represents the potential distribution of *C. cordatum* in Chiburijima. The output ASCII file (.ASC) was imported into ArcGIS 10.1 software (Licensed) to obtain the spatial distribution map of *C. cordatum*. The final map was classified using the natural breaks classification method (Jenks) [50], where higher values indicate a high degree of species existence, and lower values indicate a low degree of species adaptation.

The ROC curve was used to validate the accuracy of the final habitat suitability map. It is graphically represented by the cumulative percentage of suitability classes on the X-axis and the cumulative percentage of the training set within those classes on the Y-axis [56]. The ROC curve is an efficient method for modeling the prediction of species distribution, because of its graphical representation of the probability of true positive versus false positive [57]. The prediction accuracy of the MaxEnt model was represented by AUC.

### 3.13. Statistical Tests

Mean values and standard deviations were calculated. The Pearson’s correlation between soil, environmental parameters, and plant biological activity were measured with analysis of variance (ANOVA), calculated using XLSTAT statistical and data analysis solution (Addinsoft, 2020, New York, NY, USA). Correlations with *p*-value < 0.05 were regarded as significant.

## 4. Conclusions

In this study, the effects of environmental factors on the ACE inhibitory and antioxidant activity of *C. cordatum* were analyzed. Additionally, the habitat suitability of the species was predicted with 324 occurrence records and 32 effective conditioning factors of the study area. Most of the plant samples showed strong ACE inhibitory activity and weak radical scavenging activity. ANOVA showed that BIO05, silt, and nitrate–nitrogen significantly affected ACE inhibitory activity, while OCC significantly affected antioxidant activity. No significant correlation was found between ACE inhibitory activity and antioxidant activity of the samples. The results suggest that both activities might be more influenced by soil parameters than other environmental factors. Therefore, in the future, it will be necessary to use a variety of parameters to examine the effects of soil and the surrounding environment in more detail and to examine the differences in activity in a wide range of areas and the correlations among these parameters, in order to obtain *C. cordatum* with high biological activity and bioactive content and to preserve this plant’s distribution sites. Samples that possessed high ACE inhibition activity were mostly found in very high and high suitability areas. Climatic factors such as temperature, precipitation, annual mean temperature, and annual mean precipitation had the strongest impact on habitat suitability. Soil factors such as EC, soil bearing capacity, silt, and sand had a significant effect on habitat modeling. Topographical factors such as the digital elevation model of the study area also had a substantial impact on habitat prediction.

The obtained results of the habitat suitability map suggest that very good and good suitability areas are most appropriate for future cultivation and conservation, with specific attention paid to conditioning factors such as temperature, precipitation, BIO12, BIO01, silt, sand, soil EC, soil bearing capacity, and DEM. Based on the results of this study, it is recommended that the MaxEnt model be used to investigate other natural habitats of *C. cordatum* in Japan to obtain plants with high phytochemical content and bioactivity.

## Figures and Tables

**Table 1:** Soil macronutrients from plant collection sites.

ID ^a^	Ammonium-Nitrogen (mg 100 g^−1^) ^b^	Nitrate-Nitrogen (mg 100 g^−1^) ^b^	Available Phosphorus (mg 100 g^−1^) ^b^	Exchangeable Potassium (mg 100 g^−1^) ^b^	Exchangeable Calcium (mg 100 g^−1^) ^b^	Exchangeable Magnesium (mg 100 g^−1^) ^b^
Ch-1	2.4	0.54	68	135	398	161
Ch-2	2.4	0.54	68	135	398	161
Ch-3	2	3.1	26	47	908	136
Ch-4	2	3.1	26	47	908	136
Ch-5	2	3.1	26	47	908	136
Ch-6	1.9	2.6	37	50	720	170
Ch-7	1.9	2.6	37	50	720	170
Ch-8	2.6	0.46	7.8	39	273	157
Ch-9	2.6	0.46	7.8	39	273	157
Ch-10	1.6	1.8	76	5	523	105
Ch-11	2.5	2.6	12	46	362	133
Ch-12	2.5	2.6	12	46	362	133
Ch-13	2.6	1.4	67	33	779	202
Ch-14	2.1	6.1	47.8	97.6	138.1	97.6
Ch-15	2.1	6.1	47.8	97.6	138.1	97.6
Ch-16	5.1	6.4	38.6	65	155.1	42.2
Ch-17	5.1	6.4	38.6	65	155.1	42.2
Ch-18	5.1	6.4	38.6	65	155.1	42.2
Ch-19	2	6.5	57.2	92.4	169.8	135.5
Ch-20	2	6.5	57.2	92.4	169.8	135.5
Ch-21	2.2	1.7	37	41	552	103
Ch-22	0.8	4.2	11.5	64.7	91.9	63.1
Ch-23	0.8	4.2	11.5	64.7	91.9	63.1
Ch-24	2.4	5.1	41.2	61.7	113.8	79.5
Ch-25	2.4	5.1	41.2	61.7	113.8	79.5
Ch-26	2.4	5.1	41.2	61.7	113.8	79.5
Ch-27	1.7	2.5	25.9	70.8	99.8	119.6
Ch-28	1.7	2.5	25.9	70.8	99.8	119.6
Ch-29	3.5	2.3	41.7	41.1	209.7	105.9
Ch-30	1.9	2.9	34.5	43.7	91	86.3
Ch-31	1.9	2.9	34.5	43.7	91	86.3
Ch-32	1.9	2.9	34.5	43.7	91	86.3
Ch-33	1.8	1.6	13	49.9	96.4	56.3
Ch-34	1.8	1.6	13	49.9	96.4	56.3
Ch-35	2.4	4.7	23	40.8	117.8	138.4
Ch-36	2.4	4.7	23	40.8	117.8	138.4
Ch-37	0.9	3.8	18.3	40.4	91.5	42

^a^ Ch-no. indicates sample location. These data were analyzed from soil samples collected at distance of 0.25, 0.5, and 1 m from plants and mixed to be considered as soil sample of each location. ^b^ The percentage of content represents the mg % of dry weight.

**Table 2:** ACE inhibition activity, DPPH radical scavenging activity, and total phenolic and flavonoid content of leaf samples.

ID ^a^	ACE Inhibition Activity ^b^	DPPH Radical Scavenging Activity ^c^	Total Phenolic Content ^d^	Total Flavonoid Content ^e^
Ch-1	0.27 ± 0.09	294 ± 8.86	8.41 ± 0.25	0.48 ± 0.02
Ch-2	0.16 ± 0.02	438 ± 19.0	8.33 ± 0.15	0.17 ± 0.04
Ch-3	0.21 ± 0.01	314 ± 14.6	9.11 ± 0.20	0.39 ± 0.05
Ch-4	3.85 ± 0.67	960 ± 11.0	9.35 ± 0.11	0.13 ± 0.03
Ch-5	4.25 ± 0.04	878 ± 14.6	5.88 ± 0.17	0.14 ± 0.09
Ch-6	3.62 ± 0.44	823 ± 37.9	5.80 ± 0.10	0.38 ± 0.004
Ch-7	5.61 ± 0.03	593 ± 20.0	9.05 ± 0.25	0.15 ± 0.06
Ch-8	2.44 ± 0.53	725 ± 50.3	8.00 ± 0.05	0.13 ± 0.02
Ch-9	3.21 ± 0.28	297 ± 44.2	9.03 ± 0.12	0.55 ± 0.05
Ch-10	2.41 ± 0.33	620 ± 29.8	10.9 ± 0.12	N.D.
Ch-11	0.23 ± 0.00	650 ± 32.0	9.54 ± 0.22	0.15 ± 0.04
Ch-12	0.34 ± 0.17	630 ± 22.7	15.7 ± 0.16	0.38 ± 0.02
Ch-13	2.66 ± 0.40	889 ± 36.6	3.39 ± 0.12	0.10 ± 0.02
Ch-14	3.10 ± 0.73	369 ± 8.59	3.64 ± 0.02	0.44 ± 0.03
Ch-15	2.64 ± 0.06	404 ± 13.3	11.3 ± 0.21	1.09 ± 0.08
Ch-16	9.13 ± 0.87	517 ± 5.35	14.5 ± 0.23	0.63 ± 0.02
Ch-17	0.28 ± 0.15	466 ± 57.0	6.21 ± 0.29	0.17 ± 0.04
Ch-18	2.83 ± 0.63	760 ± 8.21	10.2 ± 0.15	0.92 ± 0.04
Ch-19	0.23 ± 0.07	338 ± 25.3	13.0 ± 0.20	1.08 ± 0.03
Ch-20	0.30 ± 0.03	1221 ± 56.6	21.7 ± 0.09	0.74 ± 0.09
Ch-21	8.74 ± 0.88	155 ± 16.5	4.35 ± 0.04	0.41 ± 0.01
Ch-22	2.16 ± 0.17	661 ± 31.8	9.57 ± 0.06	0.23 ± 0.09
Ch-23	2.79 ± 0.10	512 ± 23.6	22.6 ± 0.38	0.91 ± 0.06
Ch-24	8.80 ± 0.85	310 ± 41.4	27.1 ± 0.82	1.52 ± 0.06
Ch-25	4.50 ± 0.50	211 ± 13.2	9.87 ± 0.13	1.73 ± 0.01
Ch-26	1.88 ± 0.45	317 ± 9.70	16.6 ± 0.17	0.64 ± 0.03
Ch-27	9.60 ± 0.40	559 ± 17.7	14.9 ± 0.55	0.41 ± 0.08
Ch-28	6.18 ± 0.40	286 ± 11.1	11.7 ± 0.04	0.48 ± 0.04
Ch-29	0.64 ± 0.23	501 ± 32.4	27.6 ± 0.06	0.48 ± 0.10
Ch-30	0.41 ± 0.18	415 ± 20.5	6.83 ± 0.03	0.22 ± 0.01
Ch-31	3.46 ± 0.98	385 ± 9.31	25.5 ± 0.29	0.92 ± 0.08
Ch-32	0.41 ± 0.27	947 ± 55.8	14.1 ± 0.03	0.30 ± 0.07
Ch-33	0.53 ± 0.35	548 ± 1.84	0.91 ± 0.10	0.05 ± 0.01
Ch-34	1.29 ± 0.01	668 ± 27.0	15.6 ± 0.12	0.12 ± 0.01
Ch-35	0.77 ± 0.67	366 ± 20.8	1.38 ± 0.01	0.05 ± 0.04
Ch-36	0.22 ± 0.04	583 ± 18.4	3.32 ± 0.003	0.09 ± 0.01
Ch-37	1.10 ± 0.89	659 ± 19.4	4.75 ± 0.05	0.16 ± 0.06

Experiments were repeated three times, from which mean and standard deviation (SD) were calculated. ^a^ Ch-no. indicates sample location. ^b^ IC_50_ of ACE inhibitory activity measured in mg mL^−1^. ^c^ EC_50_ of DPPH radical scavenging activity measured in µg mL^−1^. ^d^ Total phenolic content measured as mg of gallic acid equivalent g^−1^ of extract (y = 0.0217x + 0.0561, R^2^ = 0.9673). ^e^ Total flavonoid content measured as mg of quercetin equivalent g^−1^ of extract (y = 0.0576x + 0.0099, R^2^ = 0.9996). Positive controls for ACE inhibitory experiment and DPPH radical scavenging activity were captopril (IC_50_ = 4.25 × 10^−6^ ± 5.0 × 10^−7^ mg mL^−1^) and Trolox (EC_50_ = 8.97 ± 0.80 µg mL^−1^), respectively.

**Table 3:** ANOVA of effects of variables on ACE inhibition activity and DPPH radical scavenging activity.

Variables	ACE Inhibition Activity (mg mL ^−1^)		DPPH Radical Scavenging Activity (µg mL ^−1^)	
	F Value	R^2^	F Value	R^2^
BIO05 (°C)	2.929	0.321 *	1.399	0.184
Organic Carbon Content (g/Kg)	0.583	0.783	10.986	0.986 **
Silt (%)	3.443	0.912 *	1.750	0.840
Nitrate-nitrogen (mg 100 g ^−1^)	2.455	0.637 *	1.177	0.457

* *p*-value < 0.05, ** *p*-value < 0.01.

**Table 4:** Field investigation data recorded from 37 *C. cordatum* collection sites.

ID ^a^	Latitude	Longitude	Altitude (m)	pH	Soil Bearing Capacity ^b^	SM150T Output ^c^
Ch-1	35.4332	133.0411	43.25	5.9 ± 0.79	6.7 ± 2.05	0.37 ± 0.13
Ch-2	36.0119	133.0277	30.24	6.8 ± 0.08	6.3 ± 1.25	0.28 ± 0.07
Ch-3	36.0121	133.0274	30.51	5.4 ± 0.36	8.0 ± 1.63	0.29 ± 0.01
Ch-4	36.0125	133.0268	32.7	5.5 ± 0.18	13.3 ± 1.56	0.47 ± 0.07
Ch-5	36.0131	133.0270	34.99	5.3 ± 0.19	8.9 ± 1.97	0.43 ± 0.05
Ch-6	36.0142	133.0275	50.16	5.6 ± 0.27	16.5 ± 1.18	0.84 ± 0.05
Ch-7	36.0136	133.0274	52.39	5.4 ± 0.14	12.7 ± 2.9	0.89 ± 0.05
Ch-8	36.0123	133.0285	55.53	5.4 ± 0.21	14.5 ± 0.71	0.84 ± 0.01
Ch-9	36.0119	133.0289	58.03	5.6 ± 0.5	10.8 ± 0.49	0.64 ± 0.2
Ch-10	36.0112	133.0294	60.5	6.1 ± 0.6	10.3 ± 0.8	0.68 ± 0.01
Ch-11	36.0096	133.0322	65.52	6.2 ± 0.57	7.3 ± 1.25	0.67 ± 0.02
Ch-12	36.0092	133.0325	67.66	6.3 ± 0.97	6.0 ± 0.82	0.61 ± 0.11
Ch-13	36.0171	133.0290	56.84	7.3 ± 0.33	10.3 ± 4.19	0.48 ± 0.01
Ch-14	36.0295	133.0287	50.72	6.9 ± 0.05	6.3 ± 4.03	0.56 ± 0.09
Ch-15	36.0298	133.0281	50.29	7.3 ± 0.19	10.3 ± 5.79	0.49 ± 0.09
Ch-16	36.0295	133.0220	41.35	7.0 ± 0.05	8.0 ± 2.94	0.67 ± 0.02
Ch-17	36.0297	133.0210	51.87	5.6 ± 1.02	4.7 ± 1.7	0.52 ± 0.27
Ch-18	36.0295	133.0201	51.2	7.9 ± 0.08	7.7 ± 1.7	0.37 ± 0.17
Ch-19	36.0297	133.0176	65.31	4.4 ± 0.09	8.0 ± 2.94	0.37 ± 0.07
Ch-20	36.0297	133.0167	76.78	5.6 ± 0.11	14.4 ± 0.6	0.37 ± 0.01
Ch-21	36.0051	133.0476	54.04	5.6 ± 0.00	9.0 ± 2.16	0.57 ± 0.01
Ch-22	36.0041	133.0529	39.08	4.8 ± 0.00	4.0 ± 0.00	0.5 ± 0.07
Ch-23	36.0046	133.0539	28.1	5.2 ± 0.28	12.7 ± 3.4	0.45 ± 0.03
Ch-24	36.0056	133.0593	18.78	4.8 ± 0.25	6.3 ± 2.05	0.37 ± 0.05
Ch-25	36.0061	133.0610	26.27	5.5 ± 0.31	9.7 ± 0.47	0.34 ± 0.02
Ch-26	36.0059	133.0616	32.04	5.6 ± 0.39	11.1 ± 1.6	0.28 ± 0.02
Ch-27	35.9993	133.0577	66.58	5.7 ± 0.39	7.7 ± 2.36	0.38 ± 0.16
Ch-28	35.9972	133.0584	69.96	6.6 ± 0.31	3.3 ± 0.94	0.56 ± 0.01
Ch-29	36.0114	133.0519	98.52	5.1 ± 0.5	4.3 ± 1.25	0.34 ± 0.19
Ch-30	36.0065	133.0524	63.79	5.0 ± 0.63	12.0 ± 2.16	0.24 ± 0.01
Ch-31	36.0055	133.0510	53.78	5.2 ± 0.39	6.3 ± 0.94	0.21 ± 0.03
Ch-32	36.0065	133.0512	33.32	5.0 ± 0.12	5.3 ± 0.94	0.24 ± 0.08
Ch-33	36.0151	133.0218	152.9	5.0 ± 0.14	4.7 ± 1.25	0.37 ± 0.08
Ch-34	36.0145	133.0212	175.46	6.1 ± 0.82	3.0 ± 0.00	0.37 ± 0.02
Ch-35	36.0271	133.0118	104.93	5.0 ± 0.21	5.7 ± 1.25	0.38 ± 0.01
Ch-36	36.0284	133.0150	79.36	4.9 ± 0.5	8.7 ± 1.7	0.27 ± 0.01
Ch-37	36.0188	133.0418	79.1	6.3 ± 0.19	6.7 ± 2.49	0.32 ± 0.08

^a^ ID indicates location of plant collection. ^b^ Determined in tons per square foot (t sf^−1^). ^c^ Measured in volts. All measurements were carried out in triplicate.

**Table 5:** Conditioning factors used for prediction of habitat suitability map of *C. cordatum*.

Category	Conditioning Factors	Code	Units	Data Scale
Topographic factors	Slope	Slope	°	Continuous
	Digital Elevation Model	DEM	m	Continuous
	Aspect	Aspect	°	Categorical (5 classes)
	Curvature	Curvature	m^−1^	Continuous
	Plan Curvature	Plan curvature	m^−1^	Continuous
	Profile Curvature	Profile curvature	m^−1^	Continuous
	Elevation	Elevation	m	Continuous
	Hillshade	Hillshade	m	Continuous
	Topographic Wetness Index	TWI	----	Continuous
Soil factors	pH	pH	----	Continuous
	pH in H_2_O	pH (H_2_O)	----	Continuous
	Electrical Conductivity	EC	µS/cm	Continuous
	Soil Bearing Capacity	SBC	t sf^−1^	Continuous
	Soil Moisture Sensor Output (V)	SM150T output	volts	Continuous
	Soil Bulk Density	SBD	kg/m^3^	Continuous
	Cation Exchange Capacity	CEC	cmolc/kg	Continuous
	Clay Content	Clay	%	Continuous
	Sand Content	Sand	%	Continuous
	Silt Content	Silt	%	Continuous
	Organic Carbon Density	OCD	kg/m ^3^	Continuous
	Organic Carbon Content	OCC	g/kg	Continuous
Environmental factors	Distance to Stream	----	m	Continuous
	Distance to Urban	----	m	Continuous
	Distance to Road	----	m	Continuous
Climatic factors	Precipitation	----	mm	Continuous
	Temperature	----	°C	Continuous
	Annual Mean Temperature	BIO01	°C	Continuous
	Max. Temperature of Warmest Month	BIO05	°C	Continuous
	Mini. Temperature of Coldest Month	BIO06	°C	Continuous
	Annual Precipitation	BIO12	mm/year	Continuous
	Precipitation of Wettest Month	BIO13	mm/month	Continuous
	Precipitation of Driest Month	BIO14	mm/month	Continuous

## Data Availability

Not applicable.

## Supplementary Materials

The following supporting information can be downloaded at: https://www.mdpi.com/article/10.3390/molecules27238126/s1. Table S1. Various conditional factors of plant collection sites for statistical analysis; Table S2. ANOVA of ACE inhibitory and antioxidant activity of *C. cordatum* with various conditioning factors of study area; Figure S1. Photographs of *C. cordatum* sample collection; Figure S2. Thematic maps of various conditioning factors with plant locations used for predicting habitat suitability of *C. cordatum*.
