# Supplementary material for: Phytochemical Analysis and Habitat Suitability Mapping of Cardiocrinum cordatum (Thunb.) Makino Collected at Chiburijima, Oki Islands, Japan
Source: Molecules. 2022 Nov 22;27(23):8126. doi: 10.3390/molecules27238126 (PMC9738860; doi:10.3390/molecules27238126)
Supplement: Supplementary file 1 [file molecules-27-08126-s001.zip › molecules-1874341-supplementary.pdf]

Supplementary Materials

Phytochemical Analysis and Habitat Suitability Mapping of *Cardiocrinum cordatum* (Thunb.) Makino Collected at Chiburijima, Oki Islands, Japan

Fuzuki Momotomi, Aedla Raju*, Dongxing Wang, Doaa H. M. Alsaadi and Takashi Watanabe

Supplementary Figure S1. The photographs of *C. cordatum* samples collection (at the collection sites)

Location	Longitude (E)	Latitude (N)	Elevation (m)
Ch-1	133°02'27.946"	35°25'59.423"	43.25
Ch-2	133°01'39.562"	36°00'42.822"	30.24
Ch-3	133°01'38.611"	36°00'43.564"	30.51
Ch-4	133°01'36.494"	36°00'44.824"	32.70
Ch-5	133°01'37.157"	36°00'47.232"	34.99
Ch-6	133°01'38.935"	36°00'51.080"	50.16
Ch-7	133°01'38.813"	36°00'48.913"	52.39
Ch-8	133°01'42.514"	36°00'44.348"	55.53
Ch-9	133°01'44.029"	36°00'42.962"	58.03
Ch-10	133°01'45.984"	36°00'40.478"	60.50
Ch-11	133°01'56.078"	36°00'34.448"	65.52
Ch-12	133°01'57.036"	36°00'32.929"	67.66
Ch-13	133°01'44.285"	36°01'01.582"	56.84
Ch-14	133°01'43.147"	36°01'46.193"	50.72
Ch-15	133°01'41.311"	36°01'47.323"	50.29
Ch-16	133°01'19.315"	36°01'46.074"	41.35
Ch-17	133°01'15.557"	36°01'46.952"	51.87
Ch-18	133°01'12.382"	36°01'46.279"	51.20
Ch-19	133°01'03.288"	36°01'46.970"	65.31
Ch-20	133°01'00.034"	36°01'46.808"	76.78
Ch-21	133°02'51.461"	36°00'18.367"	54.04
Ch-22	133°03'10.386"	36°00'14.706"	39.08
Ch-23	133°03'13.885"	36°00'16.578"	28.10
Ch-24	133°03'33.365"	36°00'20.246"	18.78
Ch-25	133°03'39.452"	36°00'22.097"	26.27
Ch-26	133°03'41.857"	36°00'21.262"	32.04
Ch-27	133°03'27.630"	35°59'57.336"	66.58
Ch-28	133°03'30.283"	35°59'50.039"	69.96
Ch-29	133°03'07.013"	36°00'41.155"	98.52

Ch-30	133°03'08.795"	36°00'23.497"	63.79
Ch-31	133°03'03.575"	36°00'19.901"	53.78
Ch-32	133°03'04.486"	36°00'23.454"	33.32
Ch-33	133°01'18.476"	36°00'54.209"	152.90
Ch-34	133°01'16.410"	36°00'52.182"	175.46
Ch-35	133°00'42.624"	36°01'37.434"	104.93
Ch-36	133°00'53.968"	36°01'42.096"	79.36
Ch-37	133°02'30.613"	36°01'07.745"	79.10

[a] ID represents the location of *C. cordatum* collection in which 'Ch' refers to Chiburijima Island.

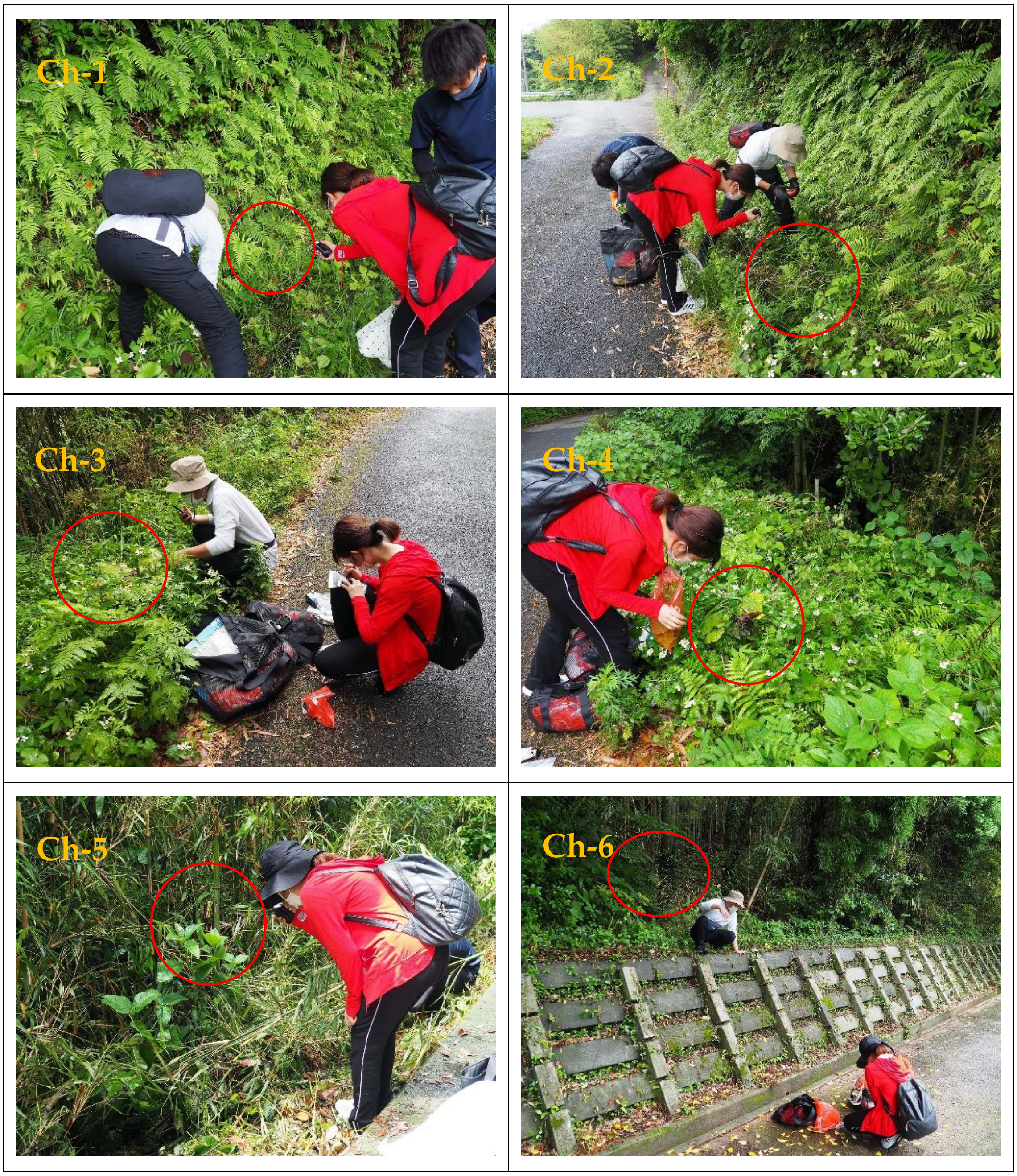

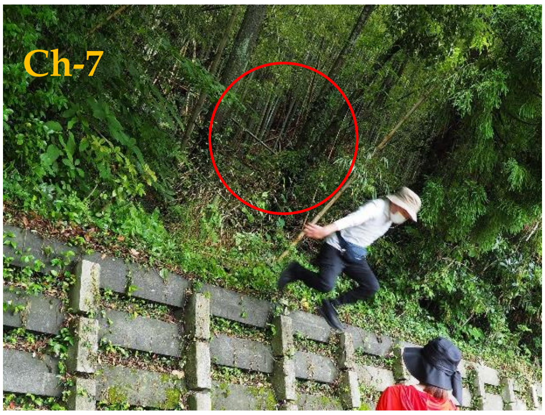
Ch-7

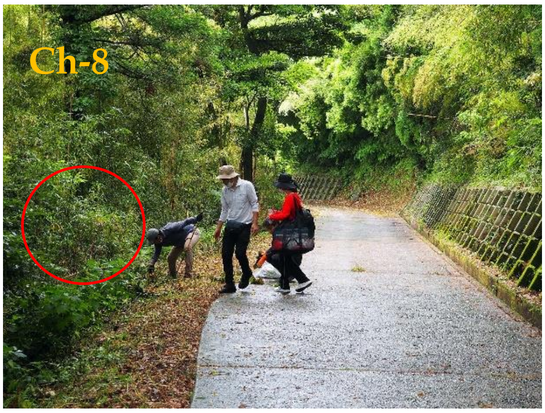
Ch-8

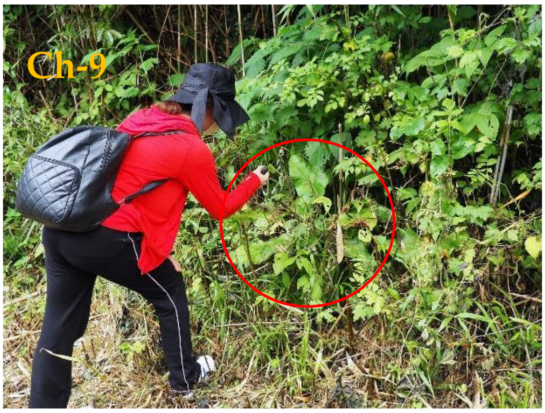
Ch-9

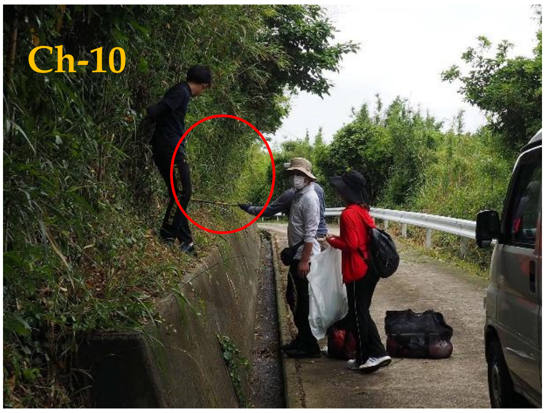
Ch-10

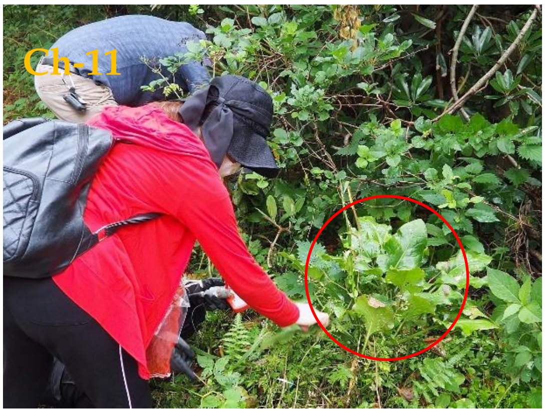
Ch-11

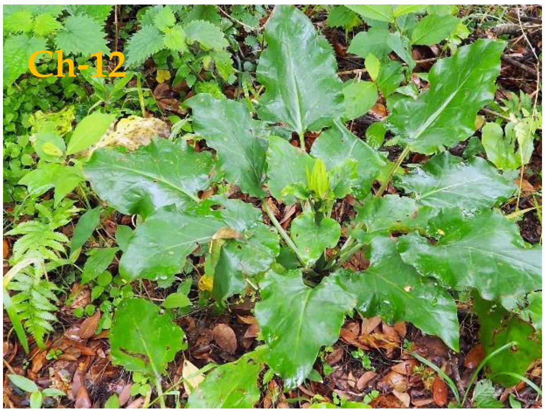
Ch-12

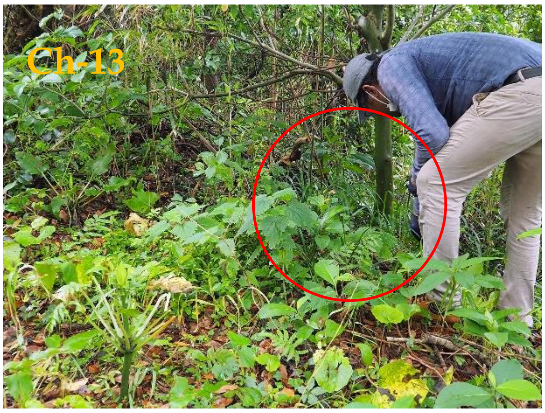
Ch-13

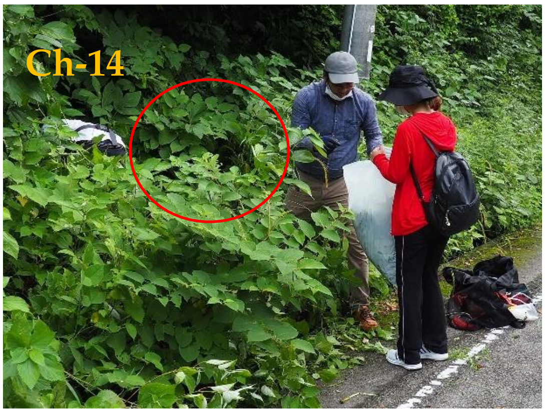
Ch-14

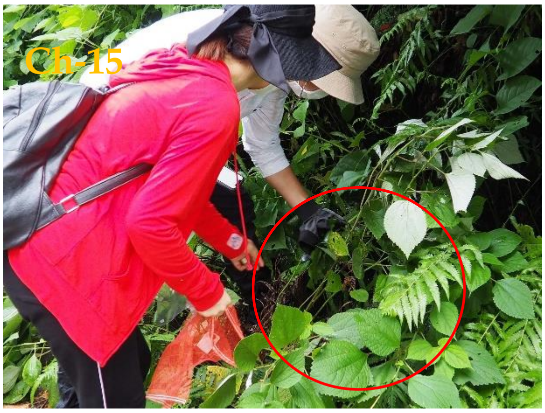Ch-15	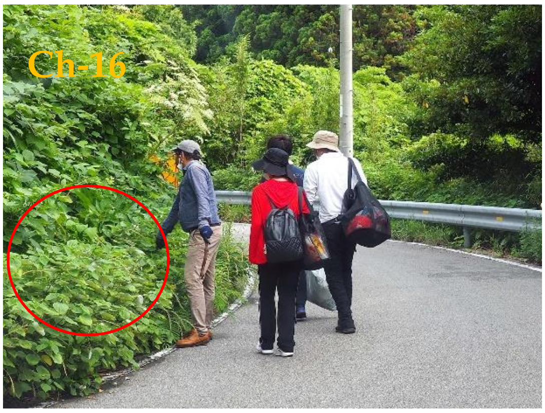Ch-16
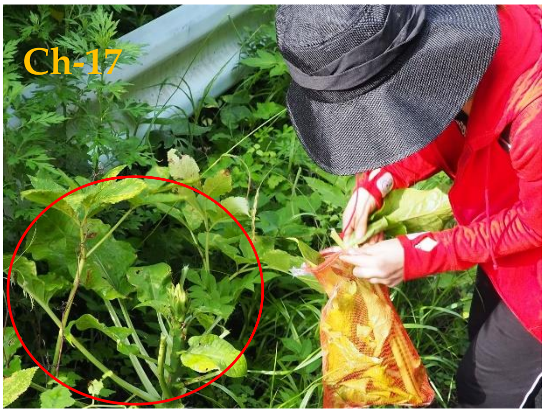Ch-17	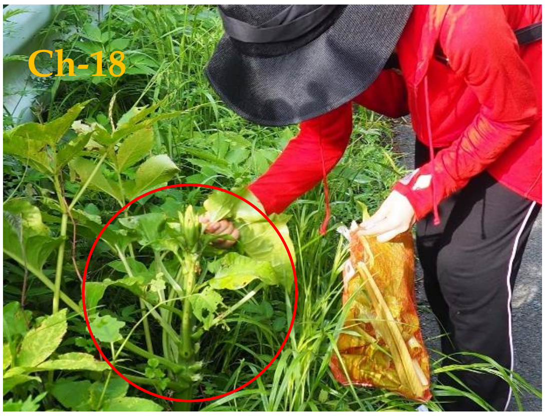Ch-18
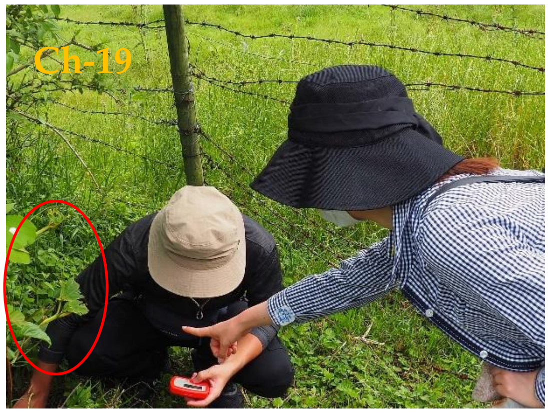Ch-19	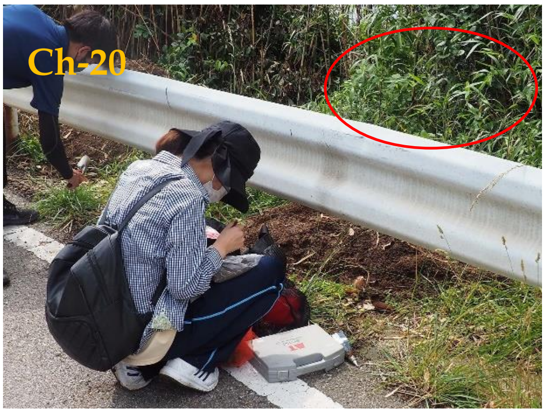Ch-20
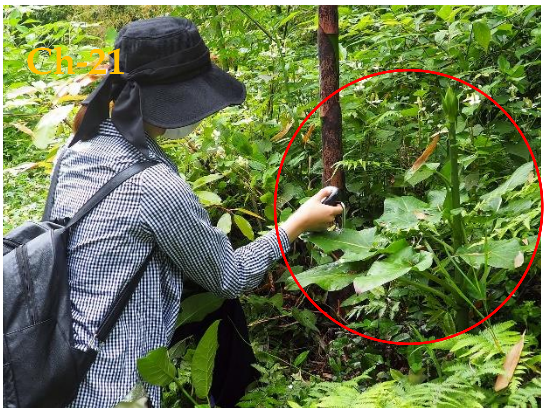Ch-21	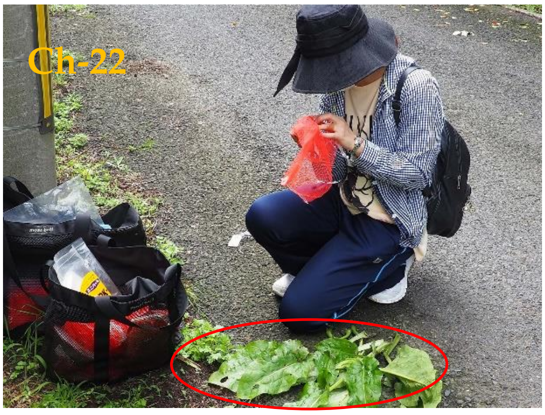Ch-22

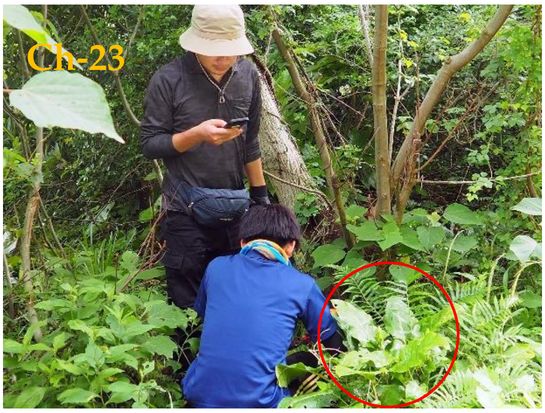
Ch-23

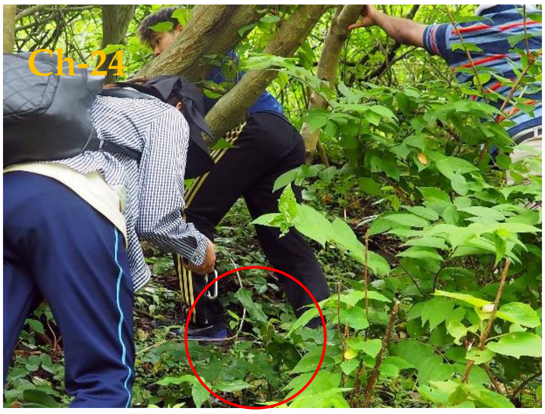
Ch-24

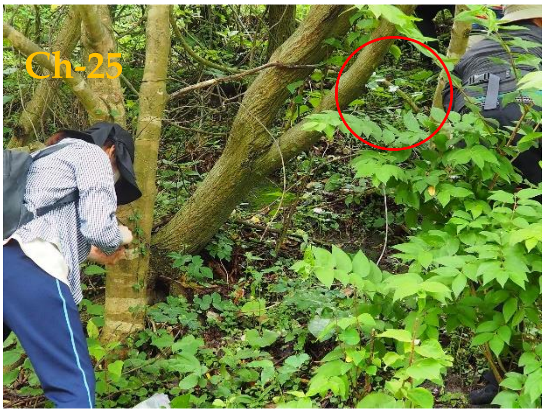
Ch-25

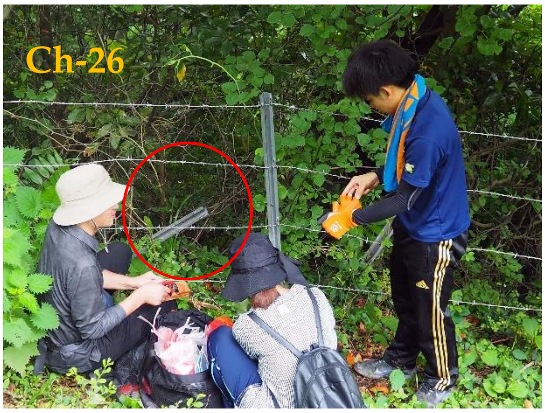
Ch-26

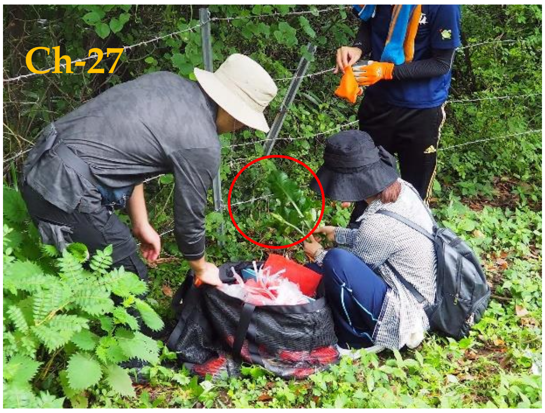
Ch-27

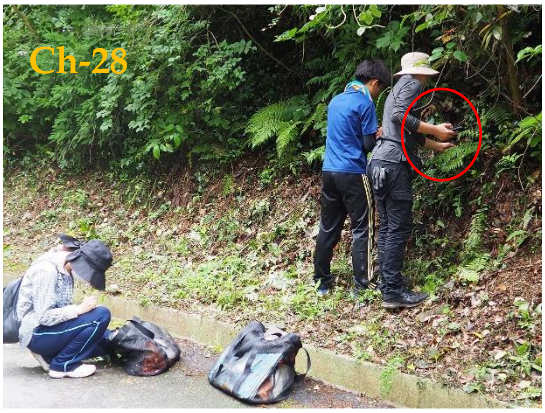
Ch-28

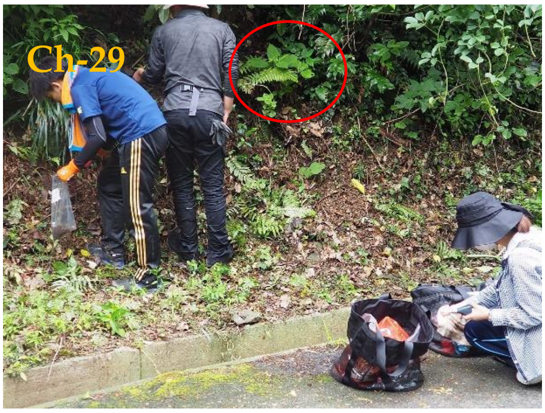
Ch-29

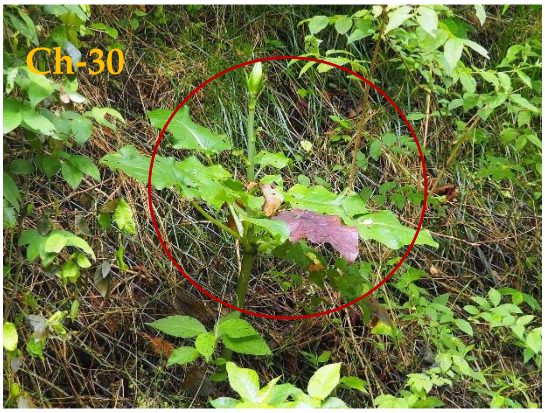
Ch-30

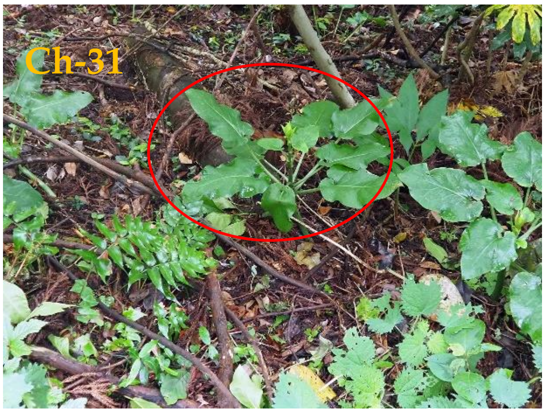
Ch-31

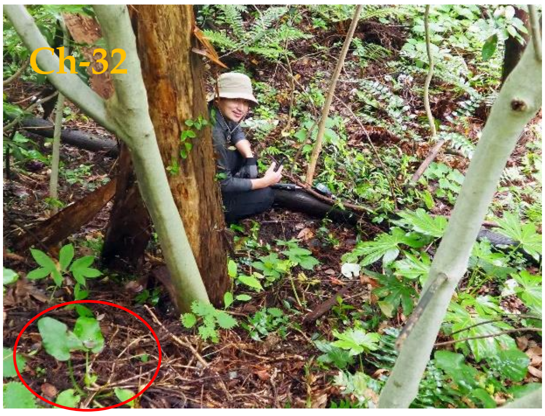
Ch-32

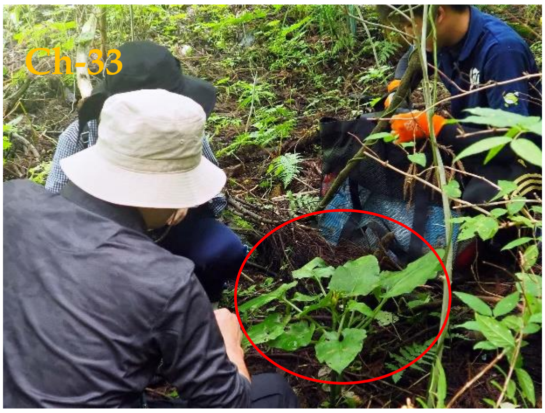
Ch-33

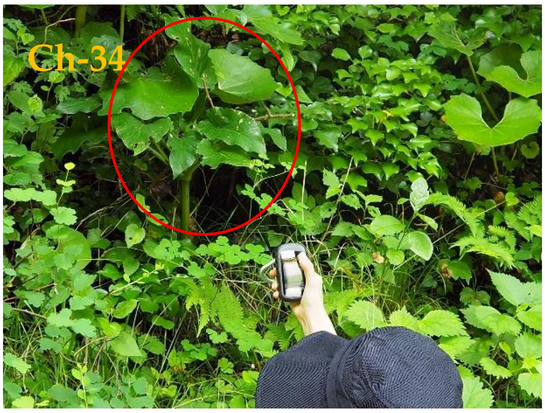
Ch-34

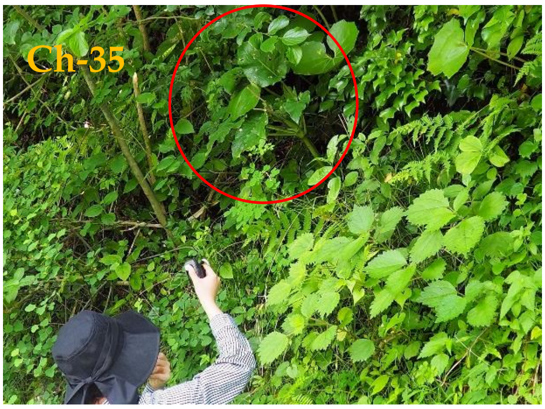
Ch-35

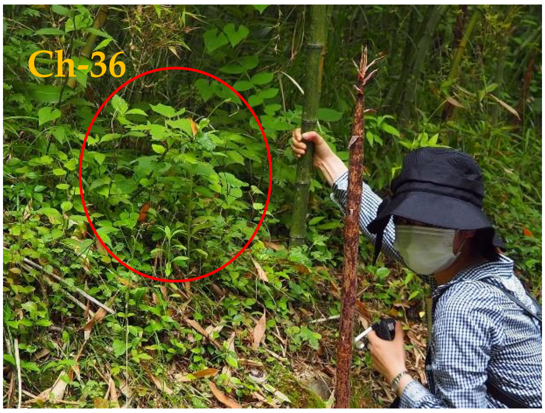
Ch-36

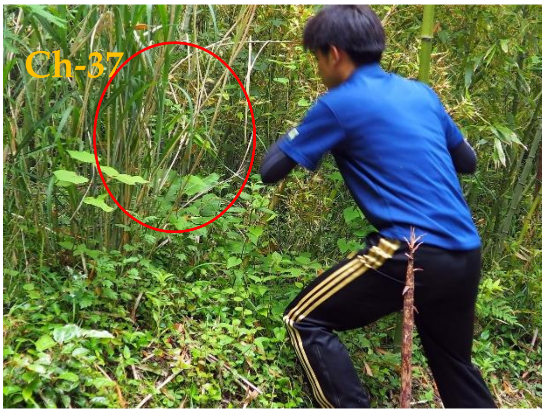
Ch-37

Supplementary Figure S2. Thematic maps of Chiburijima Island used as conditioning factors in the MaxEnt model: (**A**) Digital Eleveation Model (DEM) (m), (**B**) Slope (°), (**C**) Aspect (°), (**D**) Curvature (m^{-1}), (**E**) Plan Curvature (m^{-1}), (**F**) Profile Curvature (m^{-1}), (**G**) Hillshade (°), (**H**) Topographic Wetness Index (TWI), (**I**) Soil Cation Exchange Capacity (CEC) (cmolc/kg), (**J**) Soil Organic Carbon Content (OCC) (g/kg), (**K**) Soil Organic Carbon Density (OCD) (Kg/m^3), (**L**) Soil pH in H_2O, (**M**) Soil Bulk Density (Kg/m^3), (**N**) Soil Clay Content (%), (**O**) Soil Sand Content (%), (**P**) Soil Silt Content (%), (**Q**) Distance to Road (m), (**R**) Distance to Stream (m), (**S**) Distance to Urban (m), (**T**) Elevation (m), (**U**) pH, (**V**) Electrical Conductivity (μS/cm), (**W**) SM150T Output (V), (**X**) Soil Bearing Capacity (tsf^{-1}), (**Y**) Precipitation (mm), (**Z**) Temperature (°)

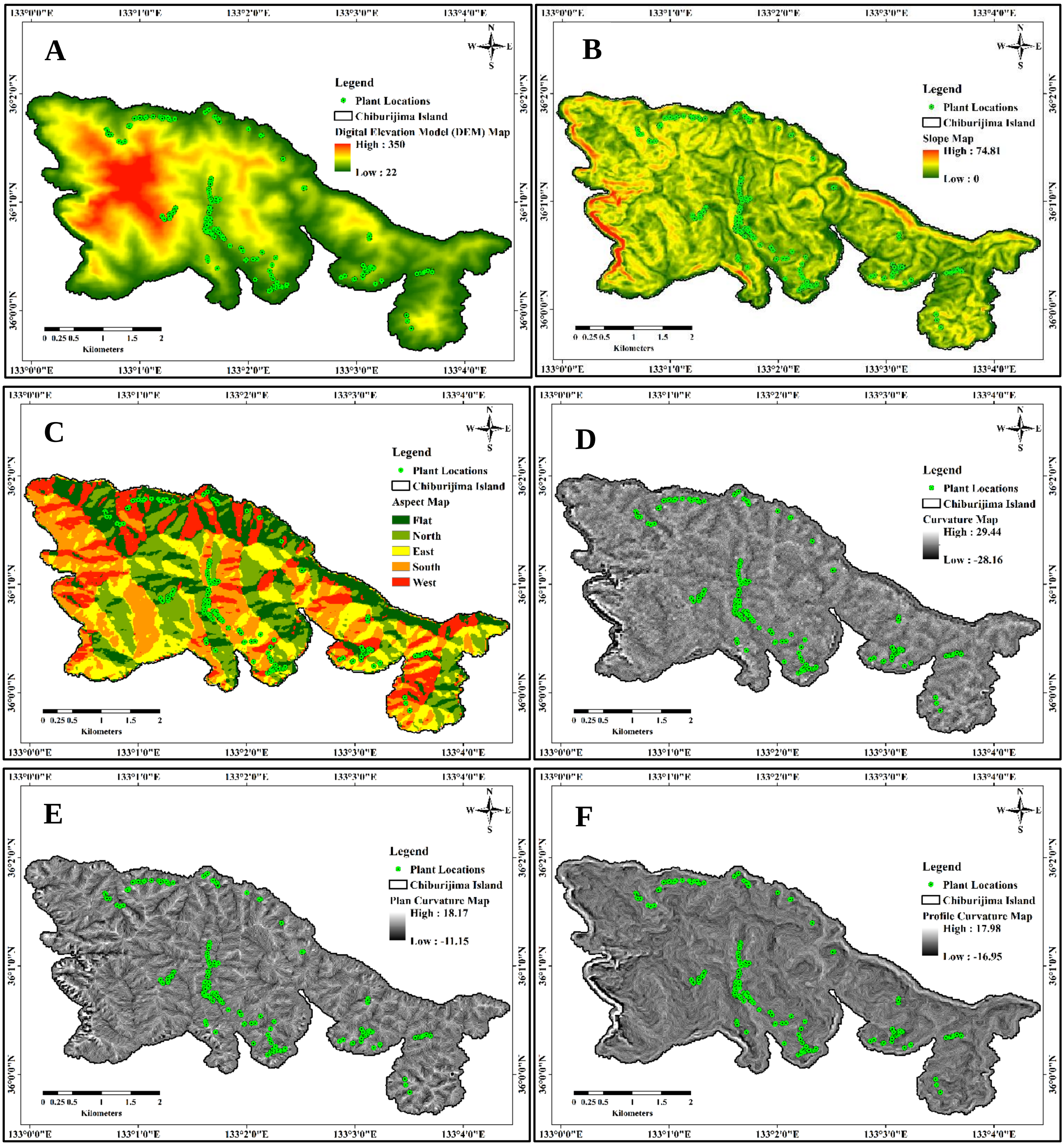

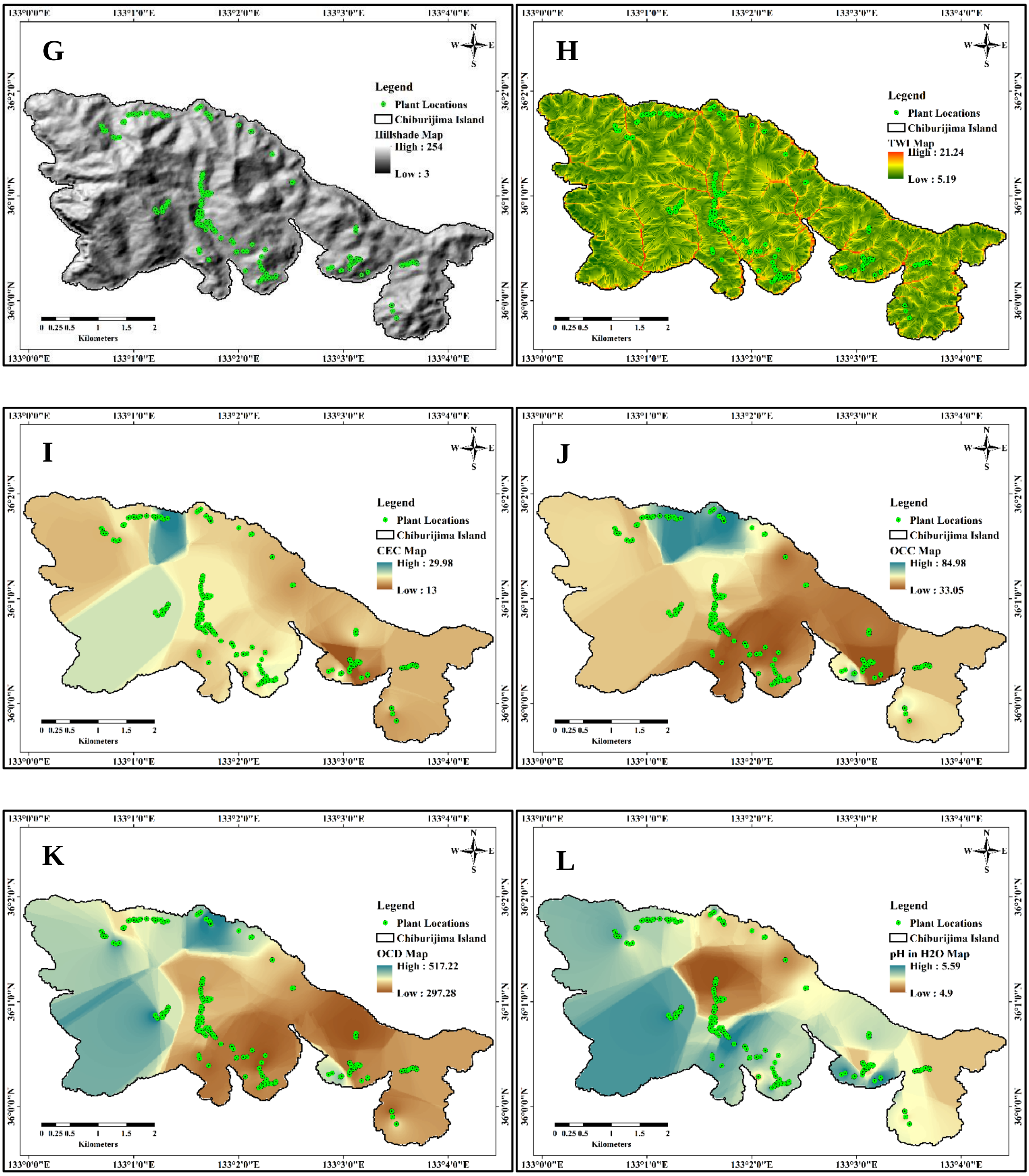
G
Legend
Plant Locations
Chiburijima Island
Hillshade Map
High : 254
Low : 3
H
Legend
Plant Locations
Chiburijima Island
TWI Map
High : 21.24
Low : 5.19
I
Legend
Plant Locations
Chiburijima Island
CEC Map
High : 29.98
Low : 13
J
Legend
Plant Locations
Chiburijima Island
OCC Map
High : 84.98
Low : 33.05
K
Legend
Plant Locations
Chiburijima Island
OCD Map
High : 517.22
Low : 297.28
L
Legend
Plant Locations
Chiburijima Island
pH in H2O Map
High : 5.59
Low : 4.9
133°0'0"E
133°1'0"E
133°2'0"E
133°3'0"E
133°4'0"E
36°2'0"N
36°1'0"N
36°0'0"N
0 0.25 0.5 1 1.5 2
Kilometers
N
W
E
S

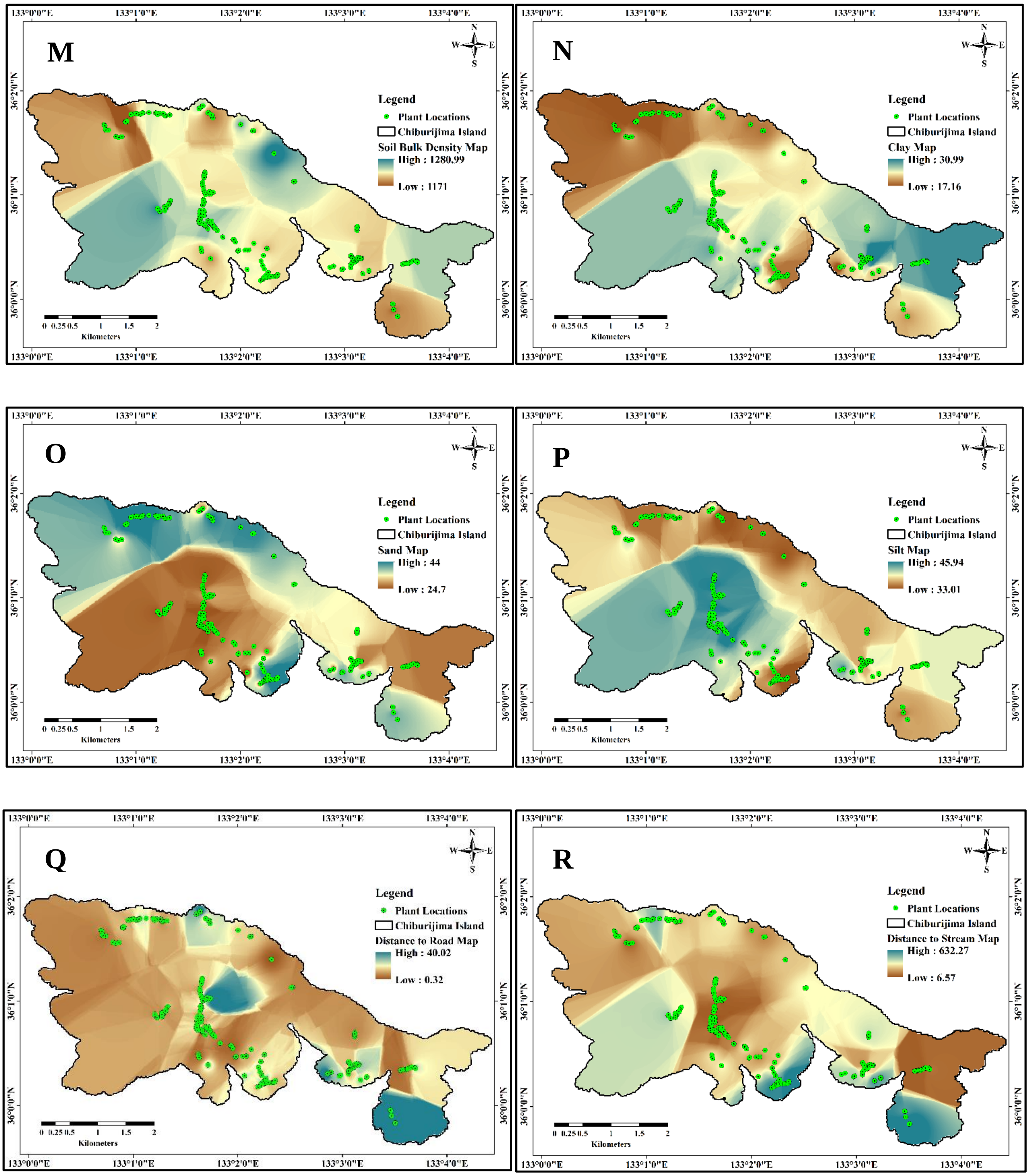

M
Legend
Plant Locations
Chiburijima Island
Soil Bulk Density Map
High : 1280.99
Low : 1171
N
Legend
Plant Locations
Chiburijima Island
Clay Map
High : 30.99
Low : 17.16
O
Legend
Plant Locations
Chiburijima Island
Sand Map
High : 44
Low : 24.7
P
Legend
Plant Locations
Chiburijima Island
Silt Map
High : 45.94
Low : 33.01
Q
Legend
Plant Locations
Chiburijima Island
Distance to Road Map
High : 40.02
Low : 0.32
R
Legend
Plant Locations
Chiburijima Island
Distance to Stream Map
High : 632.27
Low : 6.57
Kilometers
133°0'0"E
133°1'0"E
133°2'0"E
133°3'0"E
133°4'0"E
36°0'0"N
36°1'0"N
36°2'0"N

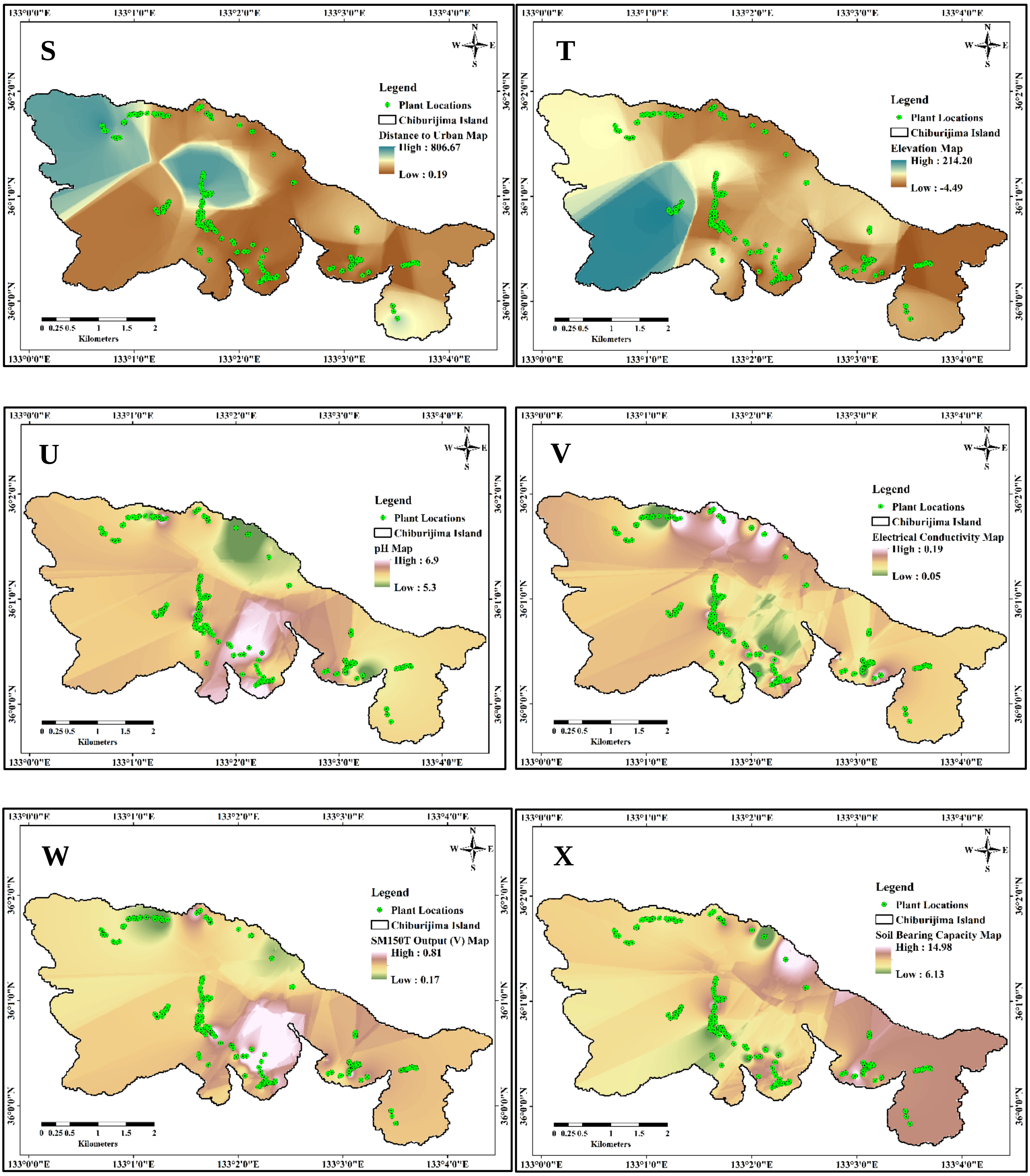
S
Legend
Plant Locations
Chiburijima Island
Distance to Urban Map
High : 806.67
Low : 0.19
T
Legend
Plant Locations
Chiburijima Island
Elevation Map
High : 214.20
Low : -4.49
U
Legend
Plant Locations
Chiburijima Island
pH Map
High : 6.9
Low : 5.3
V
Legend
Plant Locations
Chiburijima Island
Electrical Conductivity Map
High : 0.19
Low : 0.05
W
Legend
Plant Locations
Chiburijima Island
SM150T Output (V) Map
High : 0.81
Low : 0.17
X
Legend
Plant Locations
Chiburijima Island
Soil Bearing Capacity Map
High : 14.98
Low : 6.13
Kilometers

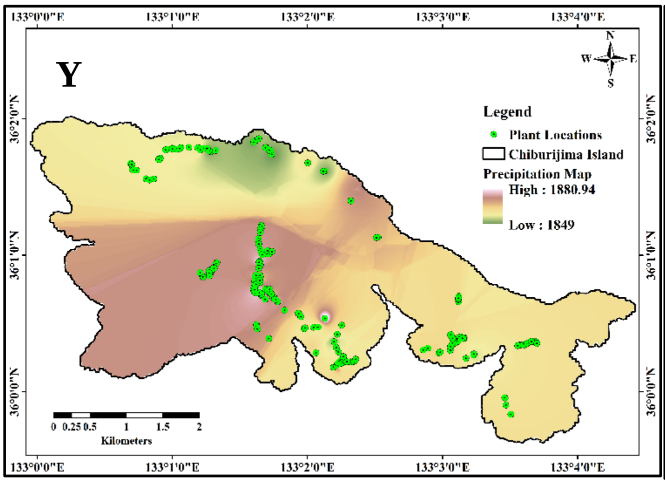
Y
Legend
Plant Locations
Chiburijima Island
Precipitation Map
High : 1880.94
Low : 1849
0 0.25 0.5 1 1.5 2
Kilometers
133°0'0"E
133°1'0"E
133°2'0"E
133°3'0"E
133°4'0"E
36°2'0"N
36°1'0"N
36°0'0"N

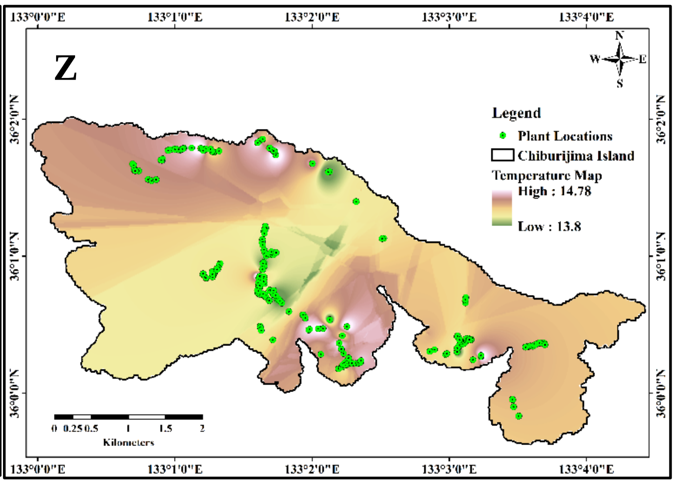
Z
Legend
Plant Locations
Chiburijima Island
Temperature Map
High : 14.78
Low : 13.8
0 0.25 0.5 1 1.5 2
Kilometers
133°0'0"E
133°1'0"E
133°2'0"E
133°3'0"E
133°4'0"E
36°2'0"N
36°1'0"N
36°0'0"N

Supplementary Table S1. Various conditional factors of the plant collection sites for statistical analysis

Sl. No.	Longitude	Latitude	GPS	Elevation (m)	Soil Bearing Capacity (ts/f)	SM150T output (V)	pH	Electrical Conductivity (EC) (mS/cm)	Nitrogen (mg/100g)	Ammonium-nitrogen (NH4+) (mg/100 g)	Nitrate – nitrogen (NO3) (mg/100 g)	Available Phosphorus (P) (mg/100 g)	Exchangeable Potassium (mg/100 g)	Exchangeable Calcium (Ca2+) (mg/100 g)	Exchangeable Magnesium (mg/100 g)
1	133.041096	35.433173	552	43.25	9.21	0.48	6.09	0.13	2.94	2.4	0.54	68	135	398	161
2	133.027656	36.011895	553	30.24	10.27	0.31	5.97	0.12	2.94	2.4	0.54	68	135	398	161
3	133.027392	36.012101	554	30.51	9.44	0.33	6.04	0.12	5.1	2	3.1	26	47	908	136
4	133.026804	36.012451	555	32.70	9.83	0.35	6.13	0.13	5.1	2	3.1	26	47	908	136
5	133.026988	36.01312	556	34.99	10.72	0.36	6.24	0.13	5.1	2	3.1	26	47	908	136
6	133.027482	36.014189	557	50.16	11.24	0.34	6.41	0.13	4.5	1.9	2.6	37	50	720	170
7	133.027448	36.013587	558	52.39	10.79	0.34	6.34	0.13	4.5	1.9	2.6	37	50	720	170
8	133.028476	36.012319	559	55.53	11.52	0.38	6.23	0.13	3.06	2.6	0.46	7.8	39	273	157
9	133.028897	36.011934	561	58.03	13.94	0.65	6.22	0.09	3.06	2.6	0.46	7.8	39	273	157
10	133.02944	36.011244	562	60.50	9.58	0.57	6.69	0.08	3.4	1.6	1.8	76	5	523	105
11	133.032244	36.009569	564	65.52	11.29	0.44	6.56	0.12	5.1	2.5	2.6	12	46	362	133
12	133.03251	36.009147	565	67.66	11.28	0.45	6.60	0.12	5.1	2.5	2.6	12	46	362	133
13	133.028968	36.017106	567	56.84	12.18	0.36	6.27	0.11	4	2.6	1.4	67	33	779	202
14	133.028652	36.029498	568	50.72	12.17	0.35	6.26	0.15	8.2	2.1	6.1	47.8	97.6	138.1	97.6
15	133.028142	36.029812	569	50.29	11.65	0.40	6.21	0.15	8.2	2.1	6.1	47.8	97.6	138.1	97.6
16	133.022032	36.029465	570	41.35	11.36	0.21	6.54	0.16	11.5	5.1	6.4	38.6	65	155.1	42.2
17	133.020988	36.029709	572	51.87	11.38	0.19	6.65	0.17	11.5	5.1	6.4	38.6	65	155.1	42.2
18	133.020106	36.029522	574	51.20	11.05	0.23	5.95	0.07	11.5	5.1	6.4	38.6	65	155.1	42.2
19	133.01758	36.029714	575	65.31	11.21	0.25	6.08	0.09	8.5	2	6.5	57.2	92.4	169.8	135.5
20	133.016676	36.029669	576	76.78	11.32	0.27	6.14	0.10	8.5	2	6.5	57.2	92.4	169.8	135.5
21	133.047628	36.005102	577	54.04	12.42	0.36	6.42	0.12	3.9	2.2	1.7	37	41	552	103
22	133.052885	36.004085	578	39.08	11.96	0.39	6.37	0.14	5	0.8	4.2	11.5	64.7	91.9	63.1
23	133.053857	36.004605	579	28.10	13.01	0.53	5.61	0.15	5	0.8	4.2	11.5	64.7	91.9	63.1
24	133.059268	36.005624	580	18.78	12.44	0.40	6.07	0.12	7.5	2.4	5.1	41.2	61.7	113.8	79.5
25	133.060959	36.006138	581	26.27	12.47	0.39	6.11	0.12	7.5	2.4	5.1	41.2	61.7	113.8	79.5
26	133.061627	36.005906	582	32.04	12.48	0.39	6.12	0.12	7.5	2.4	5.1	41.2	61.7	113.8	79.5
27	133.057675	35.99926	583	66.58	12.47	0.39	6.13	0.12	4.2	1.7	2.5	25.9	70.8	99.8	119.6
28	133.058412	35.997233	584	69.96	12.51	0.38	6.16	0.12	4.2	1.7	2.5	25.9	70.8	99.8	119.6
29	133.051948	36.011432	585	98.52	12.45	0.40	6.33	0.11	5.9	3.5	2.3	41.7	41.1	209.7	105.9
30	133.052443	36.006527	586	63.79	12.48	0.41	6.09	0.12	4.8	1.9	2.9	34.5	43.7	91	86.3
31	133.050993	36.005528	587	53.78	12.17	0.52	5.85	0.09	4.8	1.9	2.9	34.5	43.7	91	86.3
32	133.051246	36.006515	589	33.32	12.62	0.40	6.17	0.11	4.8	1.9	2.9	34.5	43.7	91	86.3
33	133.021799	36.015058	590	152.90	11.52	0.36	6.27	0.12	3.4	1.8	1.6	13	49.9	96.4	56.3
34	133.021225	36.014495	591	175.46	11.45	0.36	6.26	0.12	3.4	1.8	1.6	13	49.9	96.4	56.3
35	133.01184	36.027065	592	104.93	11.56	0.31	6.25	0.12	7.1	2.4	4.7	23	40.8	117.8	138.4
36	133.014991	36.02836	593	79.36	11.47	0.29	6.20	0.11	7.1	2.4	4.7	23	40.8	117.8	138.4
37	133.041837	36.018818	594	79.10	12.27	0.33	6.11	0.13	4.8	0.9	3.8	18.3	40.4	91.5	42

Sl. No.	Silt (%)	Sand (%)	pH in H2O	Organic Carbon Density (kg/m3)	Organic Carbon Content (g/Kg)	Clay (%)	Cation Exchange Capacity (mmol(c)/kg)	Bulk Density (Kg/m3)	Distance to Road (m)	Distance to Stream (m)	Distance to Urban (m)	Annual Mean Temperature (° C)	Annual Mean Precipitation (mm)	Slope (°)	Aspect (°)	Hillshade (°)	Curvature
1	43.00	29	5.2	355	49.00	27	20	1245	3.26	39.98	84.78	14.21	1872.45	15.79	45.00	173	-1.28
2	43.01	29	5.2	355	48.99	27	20	1245	4.86	30.85	90.37	14.16	1873.28	12.75	45.00	175	0
3	43.02	29	5.2	355	49.00	27	20	1245	7.30	30.85	122.89	14.03	1873.59	19.70	54.09	160	-1.28
4	43.21	29	5.3	373	49.37	28	20	1238	3.62	41.62	186.95	14.03	1871.39	31.87	62.20	124	0
5	43.62	29	5.2	355	51.11	27	20	1245	10.51	19.05	238.01	14.08	1867.71	8.98	124.70	150	-0.64
6	44.47	28	5	368	53.79	27	20	1235	10.04	3.61	335.54	14.09	1861.14	17.70	327.80	225	-0.64
7	44.46	29	5.2	355	53.61	27	20	1245	5.12	23.46	271.22	14.03	1859.47	15.22	233.97	181	-0.64
8	43.14	29	5.2	355	48.66	27	20	1245	1.45	54.28	123.28	13.96	1870.58	22.35	228.95	171	1.92
9	43.07	29	5.2	355	48.62	27	20	1245	0.05	72.91	94.73	13.83	1855.51	14.37	231.34	179	0.64
10	44.64	30	5.5	329	38.50	25	19	1234	1.31	76.18	65.75	13.90	1872.64	16.70	233.13	180	1.92
11	41.47	30	5.5	316	39.15	26	18	1225	1.72	210.91	76.92	14.20	1861.06	14.20	198.43	155	0
12	41.00	32	5.3	334	38.25	27	20	1230	0.88	199.21	58.12	14.27	1859.22	13.87	248.63	192	0
13	44.30	32	5	363	53.06	25	20	1219	43.48	26.26	619.77	14.02	1868.63	11.12	255.26	194	-1.28
14	32.78	44	5.1	517	82.43	23	19	1192	12.14	215.92	210.43	14.35	1849.79	19.49	42.71	172	0.64
15	32.31	44	5.1	517	79.14	23	19	1192	9.25	204.82	163.93	14.28	1850.84	21.02	51.34	161	0.64
16	36.28	41	5.2	424	79.13	22	30	1228	8.60	111.88	68.52	14.15	1851.10	22.87	31.43	182	-1.28
17	36.86	41	5.2	424	79.88	22	30	1228	3.80	200.26	159.43	14.12	1850.40	24.06	34.05	178	1.92
18	35.25	44	5.3	401	79.03	21	19	1233	0.67	245.99	214.73	14.36	1856.78	25.22	356.35	220	-1.92
19	35.27	44	5.3	408	77.80	21	19	1231	10.57	387.95	435.55	14.30	1855.45	23.32	356.01	219	-1.28
20	35.39	44	5.3	408	77.03	21	19	1231	2.62	308.39	513.31	14.29	1855.38	21.26	25.91	189	0.64
21	33.06	44	5.3	408	32.99	-9999	-9999	-9999	31.74	326.52	38.61	14.14	1859.35	12.96	272.49	205	-1.28
22	38.49	38	5.5	341	35.80	24	15	1215	13.79	445.75	27.24	14.15	1857.27	19.91	173.66	121	1.28
23	38.98	38	5.5	341	35.99	24	15	1215	7.63	404.47	23.75	14.40	1855.93	19.60	128.16	109	-1.28
24	36.30	31	5.2	346	34.82	29	18	1231	0.61	26.66	17.81	14.19	1857.48	6.50	307.87	199	0
25	35.99	30	5.1	352	34.69	30	18	1242	2.37	18.45	113.01	14.18	1857.83	2.29	0.00	185	-0.64
26	35.93	30	5.1	352	34.66	30	18	1242	11.79	71.89	174.27	14.17	1857.90	10.14	333.43	207	0
27	35.96	38	5.2	318	34.62	25	19	1201	24.16	418.08	323.99	14.17	1857.81	22.35	311.05	235	0.64
28	35.67	40	5.2	384	34.49	23	17	1199	20.87	633.93	553.21	14.16	1858.12	15.02	243.43	188	-0.64
29	33.76	35	5.3	310	33.69	28	20	1215	3.93	288.78	297.63	14.11	1859.45	23.24	297.76	233	0.64
30	35.81	30	5.2	339	34.78	31	17	1211	17.10	184.97	150.98	14.13	1857.97	19.47	331.26	227	0
31	35.62	36	5.1	326	35.86	28	13	1228	3.82	243.66	143.08	13.95	1858.47	13.61	321.71	217	0.64
32	34.84	36	5.1	326	34.40	28	13	1228	8.37	140.03	60.20	14.08	1858.85	15.28	293.75	218	-1.92
33	43.83	30	5.4	411	51.71	28	22	1240	2.60	235.11	193.99	14.07	1868.42	15.79	81.87	144	-1.28
34	43.77	30	5.4	411	51.54	28	22	1240	5.32	265.01	130.23	14.07	1868.44	20.24	77.47	135	-0.64
35	36.47	41	5.4	423	73.11	21	18	1204	0.76	141.69	714.23	14.24	1856.05	26.37	41.73	166	0
36	35.62	44	5.3	381	75.47	21	20	1171	5.28	146.72	653.23	14.27	1855.49	25.03	350.13	225	0
37	36.39	37	5.2	347	47.33	25	17	1240	5.24	231.70	23.20	14.08	1863.65	8.21	146.31	153	-0.64

Sl. No.	Profile Curvature	TWI	DEM	BIO01	BIO05	BIO06	BIO12	BIO13	BIO14
1	0.96	8.96	65	13.9	29.5	2	1875	226	116
2	0.32	9.30	63	13.9	29.5	2	1875	226	116
3	1.396757	8.74	66	13.9	29.5	2	1875	226	116
4	0.137846	7.76	78	13.9	29.5	2	1875	226	116
5	0.590769	7.45	66	13.9	29.5	2	1875	226	116
6	0.896	8.96	70	13.9	29.5	2	1875	226	116
7	0.702439	7.61	73	13.9	29.5	2	1875	226	116
8	-1.161062	8.47	76	13.9	29.5	2	1875	226	116
9	-0.390244	12.22	78	13.9	29.5	2	1875	226	116
10	-1.017705	9.22	83	13.9	29.5	2	1875	226	116
11	0	12.62	90	13.9	29.5	2	1875	226	116
12	-0.096	10.01	84	13.9	29.5	2	1875	226	116
13	0.578462	12.65	86	13.7	29.3	0	1883	228	116
14	-0.368941	8.46	73	14.4	29.9	8	1849	224	111
15	-0.685176	7.98	81	14.4	29.9	8	1849	224	111
16	0.64	9.07	61	14.4	29.9	7	1850	224	112
17	-0.984615	6.45	75	14.4	29.9	7	1850	224	112
18	1.302069	9.29	86	14.4	29.9	7	1850	224	112
19	0.611148	9.62	89	14.4	29.9	7	1850	224	112
20	0.204536	6.58	101	14.4	29.9	7	1850	224	112
21	1.28	9.28	48	14.4	29.9	7	1850	224	112
22	-0.569756	7.75	60	14.4	30	7	1858	224	113
23	0.64	7.76	51	14.4	30	7	1858	224	113
24	0.48	8.47	40	14.2	29.8	6	1867	225	114
25	0.64	12.83	53	14.2	29.8	6	1867	225	114
26	-0.256	8.43	59	14.2	29.8	6	1867	225	114
27	-0.118938	7.63	92	14.4	29.8	6	1865	225	114
28	-0.128	11.01	96	14.4	29.8	6	1854	224	113
29	-0.128	6.49	120	14.4	30	7	1854	224	112
30	0.384	6.67	96	14.4	30	7	1858	224	113
31	-0.234146	8.13	87	14.4	30	7	1858	224	113
32	1.024	9.86	72	14.4	30	7	1858	224	113
33	0.7296	8.27	183	13.5	29.1	-2	1893	231	117
34	0.745412	10.18	210	13.5	29.1	-2	1893	231	117
35	0.156098	8.84	139	13.5	29.1	0	1893	231	117
36	0	7.80	112	14.4	29.1	6	1853	224	112
37	0.443077	8.93	115	14.4	29.9	7	1853	224	112

Supplementary Table S2. ANOVA analysis of ACE inhibitory activity and antioxidant activity of C. cordatum with various conditioning factors of the study area

Summary statistics:

Variable	Observations	Obs. with missing data	Obs. without missing data	Minimum	Maximum	Mean	Std. deviation
IC50 Average（mg/ml）	37	0	37	0.160	9.600	2.736	2.763
DPPH activity IC50（μg/ml）	37	0	37	154.950	1221.360	547.826	238.704
Elevation (m)	37	0	37	18.800	175.500	59.514	32.009
Soil Bearing Capacity (ts/f)	37	0	37	9.210	13.940	11.600	1.026
SM150T output (V)	37	0	37	0.190	0.650	0.375	0.093
pH	37	0	37	5.610	6.690	6.221	0.219
Electrical Conductivity (EC) (mS/cm)	37	0	37	0.070	0.170	0.121	0.020
Nitrogen (mg/100g)	37	0	37	2.900	11.500	5.749	2.400
Ammonium-nitrogen (NH4+) (mg/100 g)	37	0	37	0.800	5.100	2.308	0.984
Nitrate –nitrogen (NO3) (mg/100 g)	37	0	37	0.500	6.500	3.435	1.902
Available Phosphorus (P) (mg/100 g)	37	0	37	7.800	76.000	34.251	18.237
Exchangeable Potassium (mg/100 g)	37	0	37	5.000	135.000	59.057	26.395
Exchangeable Calcium (Ca2+) (mg/100 g)	37	0	37	91.000	908.000	294.392	267.899
Exchangeable Magnesium (mg/100 g)	37	0	37	42.000	202.000	109.538	42.464
Silt (%)	37	0	37	32.300	44.600	38.738	4.087
Sand (%)	37	0	37	28.000	44.000	34.973	5.960
pH in H2O	37	0	37	5.000	5.500	5.243	0.132
Organic Carbon Density (kg/m3)	37	0	37	310.000	517.000	372.514	47.929
Organic Carbon Content (g/Kg)	37	0	37	33.000	82.400	51.124	16.966
Clay (%)	37	0	37	-9999.000	31.000	-245.297	1648.043
Cation Exchange Capacity (mmol(c)/kg)	37	0	37	-9999.000	30.000	-251.486	1646.998
Bulk Density (Kg/m3)	37	0	37	-9999.000	1245.000	923.081	1845.546
Distance to Road (m)	37	0	37	0.100	43.500	8.292	9.187
Distance to Stream (m)	37	0	37	3.610	633.940	181.834	148.907
Distance to Urban (m)	37	0	37	17.810	714.230	210.079	188.744
Annual Mean Temperature (° C)	37	0	37	13.800	14.400	14.151	0.137

Annual Mean Precipitation (mm)	37	0	37	1849.800	1873.600	1860.681	6.906
Slope (°)	37	0	37	2.290	31.870	17.511	6.157
Aspect (°)	37	0	37	0.000	356.350	186.444	120.129
Hillshade (°)	37	0	37	109.000	235.000	182.351	32.688
Curvature	37	0	37	-1.920	1.920	-0.190	1.022
Plan Curvature	37	0	37	-0.900	0.940	0.045	0.499
Profile Curvature	37	0	37	-1.160	1.400	0.235	0.651
TWI	37	0	37	6.450	12.830	8.913	1.659
DEM	37	0	37	40.000	210.000	85.865	33.995
BIO01	37	0	37	13.500	14.400	14.130	0.304
BIO05	37	0	37	29.100	30.000	29.670	0.289
BIO06	37	0	37	-2.000	8.000	4.405	2.976
BIO12	37	0	37	-9999.000	1893.000	262.703	4112.263
BIO13	37	0	37	-9999.000	231.000	-1156.270	3543.611
BIO14	37	0	37	-9999.000	117.000	-1252.432	3505.075

Correlation matrix (Pearson):

Variables+B57:AH57	IC50 Average (mg/ml)	DPPH activity IC50 (μg/ml)	Elevation (m)	Soil Bearing Capacity (ts/f)	SM150T output (V)	pH	Electrical Conductivity (EC) (mS/cm)	Nitrogen (mg/100g)	Ammonium-nitrogen (NH4+) (mg/100 g)	Nitrate – nitrogen (NO3) (mg/100 g)	Available Phosphorus (P) (mg/100 g)	Exchangeable Potassium (mg/100 g)	Exchangeable Calcium (Ca2+) (mg/100 g)	Exchangeable Magnesium (mg/100 g)
IC50 Average (mg/ml)	**1**	-0.202	-0.282	0.262	0.014	0.028	0.144	0.069	0.075	0.050	-0.020	-0.075	0.072	-0.092
DPPH activity IC50 (μg/ml)	-0.202	**1**	0.099	-0.210	-0.210	0.121	-0.117	-0.018	-0.045	0.000	-0.040	-0.201	0.271	0.153
Elevation (m)	-0.282	0.099	**1**	0.058	-0.087	0.206	-0.164	-0.185	-0.073	-0.199	-0.281	-0.215	-0.282	-0.142
Soil Bearing Capacity (ts/f)	0.262	-0.210	0.058	**1**	0.250	-0.189	0.003	0.040	-0.094	0.098	**-0.361**	-0.204	**-0.564**	-0.278
SM150T output (V)	0.014	-0.210	-0.087	0.250	**1**	-0.112	-0.278	**-0.620**	**-0.456**	**-0.546**	-0.138	-0.235	0.007	0.163
pH	0.028	0.121	0.206	-0.189	-0.112	**1**	0.183	0.076	0.301	-0.060	0.017	**-0.337**	0.201	0.058

Variables+B57:AH57	Silt (%)	Sand (%)	pH in H2O	Organic Carbon Density (kg/m3)	Organic Carbon Content (g/Kg)	Clay (%)	Cation Exchange Capacity (mmol(c)/kg)	Bulk Density (Kg/m3)	Distance to Road (m)	Distance to Stream (m)	Distance to Urban (m)	Annual Mean Temperature (° C)	Annual Mean Precipitation (mm)	Slope (°)	Aspect (°)	Hillshade (°)	Curvature	Plan Curvature	Profile Curvature	TWI	DEM	BIO01	BIO05	BIO06	BIO12	BIO13	BIO14
IC50 Average (mg/ml)	-0.164	0.103	-0.207	0.031	-0.181	**-0.367**	**-0.367**	**-0.368**	**0.358**	0.085	-0.103	-0.054	-0.221	-0.177	0.140	0.141	-0.079	-0.032	0.100	0.050	**-0.367**	0.226	0.232	0.216	**-0.343**	**-0.343**	**-0.343**
DPPH activity IC50 (μg/ml)	0.298	-0.065	0.072	-0.131	0.155	0.278	0.278	0.279	-0.034	-0.101	0.180	-0.044	0.209	0.245	0.000	-0.116	0.011	0.052	0.024	-0.157	0.178	-0.132	-0.131	-0.185	0.265	0.265	0.265
Elevation (m)	0.102	0.056	**0.367**	0.188	0.135	0.029	0.029	0.028	-0.087	0.306	0.300	-0.064	0.095	0.232	-0.057	-0.075	0.003	0.001	-0.003	-0.007	**0.968**	**-0.401**	**-0.483**	**-0.430**	-0.193	-0.193	-0.194
Soil Bearing Capacity (ts/f)	**-0.491**	0.244	-0.172	-0.043	-0.257	-0.135	-0.136	-0.138	0.276	**0.379**	0.014	0.065	**-0.599**	-0.262	**0.337**	0.225	0.034	-0.120	-0.145	0.171	0.000	**0.388**	**0.383**	**0.410**	-0.192	-0.191	-0.191
SM150T output (V)	0.259	**-0.440**	0.133	**-0.443**	**-0.622**	0.029	0.027	0.029	-0.095	-0.046	**-0.367**	**-0.380**	0.191	**-0.346**	0.175	-0.128	0.212	0.103	-0.253	0.275	-0.118	-0.162	-0.016	-0.167	0.127	0.126	0.126
pH	0.183	-0.048	0.145	0.101	0.116	-0.154	-0.153	-0.154	0.012	-0.125	-0.025	-0.259	-0.051	0.053	-0.059	0.030	**0.348**	0.280	**-0.333**	0.107	0.093	-0.217	-0.224	-0.225	-0.024	-0.025	-0.025

Electrical Conductivity (EC) (mS/cm)	0.144	-0.117	-0.164	0.003	-0.278	0.183	**1**	0.194	0.044	0.222	-0.182	0.281	-0.016	-0.199	-0.062	0.080	-0.110	**0.360**	0.176	0.009	0.010	0.007	0.098	0.002	-0.205	0.219	-0.230	0.092	**-0.522**	**-0.442**	0.104	0.028	-0.143	-0.283	-0.190	0.102	0.128	0.135	0.060	0.061	0.060
Nitrogen (mg/100g)	0.069	-0.018	-0.185	0.040	**-0.620**	0.076	0.194	**1**	**0.659**	**0.921**	0.174	0.192	-0.318	**-0.451**	**-0.582**	**0.616**	-0.097	**0.494**	**0.661**	0.129	0.131	0.128	-0.174	0.068	0.147	**0.528**	**-0.676**	0.296	-0.117	0.193	-0.010	-0.058	-0.030	-0.218	-0.164	**0.484**	**0.387**	**0.552**	0.073	0.075	0.075
Ammonium-nitrogen (NH4+) (mg/100 g)	0.075	-0.045	-0.073	-0.094	**-0.456**	0.301	0.044	**0.659**	**1**	0.314	0.178	0.065	-0.058	-0.182	-0.171	0.215	-0.169	0.231	**0.468**	0.018	0.020	0.019	-0.157	-0.208	0.036	0.090	-0.275	0.267	-0.040	0.319	-0.011	-0.029	-0.007	0.003	-0.091	0.113	0.078	0.127	0.092	0.093	0.093
Nitrate – nitrogen (NO3) (mg/100 g)	0.050	0.000	-0.199	0.098	**-0.546**	-0.060	0.222	**0.921**	0.314	**1**	0.128	0.211	**-0.370**	**-0.473**	**-0.643**	**0.665**	-0.035	**0.507**	**0.594**	0.153	0.154	0.151	-0.137	0.191	0.168	**0.621**	**-0.711**	0.237	-0.127	0.078	-0.007	-0.059	-0.034	-0.275	-0.162	**0.549**	**0.445**	**0.628**	0.043	0.045	0.045
Available Phosphorus (P) (mg/100 g)	-0.020	-0.040	-0.281	**-0.361**	-0.138	0.017	-0.182	0.174	0.178	0.128	**1**	**0.408**	0.171	0.229	-0.091	0.097	-0.321	0.162	0.220	-0.026	-0.025	-0.025	0.201	-0.249	0.157	0.067	0.108	-0.106	-0.045	**0.354**	-0.024	0.035	0.064	-0.027	-0.279	0.125	0.165	0.155	0.160	0.160	0.160
Exchangeable Potassium (mg/100 g)	-0.075	-0.201	-0.215	-0.204	-0.235	**-0.337**	0.281	0.192	0.065	0.211	**0.408**	**1**	-0.201	0.065	-0.198	0.228	-0.182	**0.370**	0.321	0.115	0.116	0.115	-0.044	0.124	-0.024	**0.558**	-0.112	0.037	**-0.375**	-0.055	-0.108	-0.099	0.094	-0.118	-0.205	0.211	0.219	0.231	0.094	0.094	0.094
Exchangeable Calcium (Ca2+) (mg/100 g)	0.072	0.271	-0.282	**-0.564**	0.007	0.201	-0.016	-0.318	-0.058	**-0.370**	0.171	-0.201	**1**	**0.610**	**0.638**	**-0.471**	-0.151	-0.159	-0.068	-0.162	-0.162	-0.159	0.198	**-0.523**	0.033	**-0.455**	**0.582**	-0.042	-0.078	-0.158	-0.155	0.034	0.270	0.076	-0.269	**-0.451**	**-0.358**	**-0.484**	0.146	0.145	0.145
Exchangeable Magnesium (mg/100 g)	-0.092	0.153	-0.142	-0.278	0.163	0.058	-0.199	**-0.451**	-0.182	**-0.473**	0.229	0.065	**0.610**	**1**	**0.470**	**-0.328**	-0.247	-0.168	-0.008	0.026	0.026	0.027	0.198	**-0.360**	**0.449**	-0.280	**0.372**	-0.008	0.112	0.155	0.035	0.072	0.001	0.229	-0.155	**-0.424**	**-0.459**	**-0.456**	-0.133	-0.134	-0.134
Silt (%)	-0.164	0.298	0.102	**-0.491**	0.259	0.183	-0.062	**-0.582**	-0.171	**-0.643**	-0.091	-0.198	**0.638**	**0.470**	**1**	**-0.777**	0.138	**-0.333**	-0.179	0.234	0.233	0.239	-0.133	**-0.489**	-0.112	**-0.542**	**0.748**	-0.107	-0.158	**-0.413**	-0.019	0.070	0.083	0.235	0.123	**-0.804**	**-0.672**	**-0.859**	**0.332**	**0.330**	**0.330**
Sand (%)	0.103	-0.065	0.056	0.244	**-0.440**	-0.048	0.080	**0.616**	0.215	**0.665**	0.097	0.228	**-0.471**	**-0.328**	**-0.777**	**1**	0.093	**0.571**	**0.557**	-0.257	-0.256	-0.262	0.187	**0.603**	**0.341**	**0.598**	**-0.691**	**0.403**	0.016	0.194	-0.018	-0.131	-0.072	-0.231	0.014	**0.646**	**0.415**	**0.660**	**-0.434**	**-0.432**	**-0.433**
pH in H2O	-0.207	0.072	**0.367**	-0.172	0.133	0.145	-0.110	-0.097	-0.169	-0.035	-0.321	-0.182	-0.151	-0.247	0.138	0.093	**1**	-0.139	-0.084	-0.073	-0.073	-0.073	-0.314	**0.391**	-0.120	0.135	0.107	0.318	-0.108	**-0.465**	0.128	0.132	-0.100	-0.102	0.323	-0.157	-0.176	-0.180	-0.111	-0.111	-0.111
Organic Carbon Density (kg/m3)	0.031	-0.131	0.188	-0.043	**-0.443**	0.101	**0.360**	**0.494**	0.231	**0.507**	0.162	**0.370**	-0.159	-0.168	**-0.333**	**0.571**	-0.139	**1**	**0.775**	-0.126	-0.124	-0.128	0.059	0.105	0.225	**0.386**	**-0.400**	0.324	**-0.438**	-0.167	-0.010	-0.100	-0.061	-0.113	0.168	0.072	-0.022	0.144	-0.087	-0.086	-0.086
Organic Carbon Content (g/Kg)	-0.181	0.155	0.135	-0.257	**-0.622**	0.116	0.176	**0.661**	**0.468**	**0.594**	0.220	0.321	-0.068	-0.008	-0.179	**0.557**	-0.084	**0.775**	**1**	0.179	0.182	0.178	-0.163	-0.069	**0.409**	**0.362**	-0.359	**0.513**	**-0.327**	-0.015	0.003	-0.044	-0.038	-0.187	0.163	0.056	-0.126	0.092	0.022	0.023	0.023
Clay (%)	**-0.367**	0.278	0.029	-0.135	0.029	-0.154	0.009	0.129	0.018	0.153	-0.026	0.115	-0.162	0.026	0.234	-0.257	-0.073	-0.126	0.179	**1**	**1.000**	**1.000**	**-0.431**	-0.165	0.153	0.063	0.032	0.124	-0.121	-0.117	0.180	0.016	-0.271	-0.037	0.188	-0.150	-0.135	-0.148	**0.422**	**0.422**	**0.422**

Cation Exchange Capacity (mmol(c)/kg)	**-0.367**	0.278	0.029	-0.136	0.027	-0.153	0.010	0.131	0.020	0.154	-0.025	0.116	-0.162	0.026	0.233	-0.256	-0.073	-0.124	0.182	**1.000**	**1**	**1.000**	**-0.431**	-0.165	0.154	0.063	0.031	0.125	-0.122	-0.117	0.180	0.016	-0.271	-0.038	0.188	-0.150	-0.135	-0.148	**0.422**	**0.422**	**0.422**
Bulk Density (Kg/m3)	**-0.368**	0.279	0.028	-0.138	0.029	-0.154	0.007	0.128	0.019	0.151	-0.025	0.115	-0.159	0.027	0.239	-0.262	-0.073	-0.128	0.178	**1.000**	**1.000**	**1**	**-0.434**	-0.169	0.149	0.059	0.037	0.121	-0.123	-0.119	0.179	0.016	-0.268	-0.036	0.187	-0.154	-0.136	-0.151	**0.427**	**0.427**	**0.427**
Distance to Road (m)	**0.358**	-0.034	-0.087	0.276	-0.095	0.012	0.098	-0.174	-0.157	-0.137	0.201	-0.044	0.198	0.198	-0.133	0.187	-0.314	0.059	-0.163	**-0.431**	**-0.431**	**-0.434**	**1**	0.259	0.281	-0.030	-0.024	-0.131	0.250	0.212	-0.232	-0.260	0.165	0.153	-0.145	0.165	0.132	0.122	**-0.361**	**-0.362**	**-0.362**
Distance to Stream (m)	0.085	-0.101	0.306	**0.379**	-0.046	-0.125	0.002	0.068	-0.208	0.191	-0.249	0.124	**-0.523**	**-0.360**	**-0.489**	**0.603**	**0.391**	0.105	-0.069	-0.165	-0.165	-0.169	0.259	**1**	0.151	**0.409**	**-0.398**	0.243	0.161	-0.004	0.018	-0.129	-0.128	-0.121	0.243	**0.489**	**0.408**	**0.431**	**-0.409**	**-0.408**	**-0.408**
Distance to Urban (m)	-0.103	0.180	0.300	0.014	**-0.367**	-0.025	-0.205	0.147	0.036	0.168	0.157	-0.024	0.033	**0.449**	-0.112	**0.341**	-0.120	0.225	**0.409**	0.153	0.154	0.149	0.281	0.151	**1**	0.147	-0.152	0.320	0.136	0.312	-0.047	-0.111	-0.011	-0.016	**0.330**	-0.098	**-0.350**	-0.113	**-0.524**	**-0.523**	**-0.524**
Annual Mean Temperature (° C)	-0.054	-0.044	-0.064	0.065	**-0.380**	-0.259	0.219	**0.528**	0.090	**0.621**	0.067	**0.558**	**-0.455**	-0.280	**-0.542**	**0.598**	0.135	**0.386**	**0.362**	0.063	0.063	0.059	-0.030	**0.409**	0.147	**1**	**-0.489**	0.155	-0.082	0.033	-0.221	-0.282	0.131	-0.152	-0.069	**0.423**	0.279	**0.460**	-0.144	-0.143	-0.143
Annual Mean Precipitation (mm)	-0.221	0.209	0.095	**-0.599**	0.191	-0.051	-0.230	**-0.676**	-0.275	**-0.711**	0.108	-0.112	**0.582**	**0.372**	**0.748**	**-0.691**	0.107	**-0.400**	**-0.359**	0.032	0.031	0.037	-0.024	**-0.398**	-0.152	**-0.489**	**1**	-0.145	-0.133	-0.291	-0.109	0.038	0.200	0.140	0.153	**-0.641**	**-0.522**	**-0.694**	0.194	0.192	0.192
Slope (°)	-0.177	0.245	0.232	-0.262	**-0.346**	0.053	0.092	0.296	0.267	0.237	-0.106	0.037	-0.042	-0.008	-0.107	**0.403**	0.318	0.324	**0.513**	0.124	0.125	0.121	-0.131	0.243	0.320	0.155	-0.145	**1**	-0.060	-0.031	0.185	0.147	-0.179	**-0.459**	0.271	0.083	-0.058	0.055	-0.185	-0.185	-0.185
Aspect (°)	0.140	0.000	-0.057	**0.337**	0.175	-0.059	**-0.522**	-0.117	-0.040	-0.127	-0.045	**-0.375**	-0.078	0.112	-0.158	0.016	-0.108	**-0.438**	**-0.327**	-0.121	-0.122	-0.123	0.250	0.161	0.136	-0.082	-0.133	-0.060	**1**	**0.685**	-0.056	-0.102	0.010	0.018	-0.095	0.273	0.170	0.215	-0.191	-0.191	-0.191
Hillshade (°)	0.141	-0.116	-0.075	0.225	-0.128	0.030	**-0.442**	0.193	0.319	0.078	**0.354**	-0.055	-0.158	0.155	**-0.413**	0.194	**-0.465**	-0.167	-0.015	-0.117	-0.117	-0.119	0.212	-0.004	0.312	0.033	-0.291	-0.031	**0.685**	**1**	-0.044	0.010	0.076	-0.038	-0.114	**0.381**	0.244	**0.355**	-0.264	-0.263	-0.263
Curvature	-0.079	0.011	0.003	0.034	0.212	**0.348**	0.104	-0.010	-0.011	-0.007	-0.024	-0.108	-0.155	0.035	-0.019	-0.018	0.128	-0.010	0.003	0.180	0.180	0.179	-0.232	0.018	-0.047	-0.221	-0.109	0.185	-0.056	-0.044	**1**	**0.853**	**-0.917**	-0.311	-0.029	0.076	0.065	0.090	0.025	0.026	0.026
Plan Curvature	-0.032	0.052	0.001	-0.120	0.103	0.280	0.028	-0.058	-0.029	-0.059	0.035	-0.099	0.034	0.072	0.070	-0.131	0.132	-0.100	-0.044	0.016	0.016	0.016	-0.260	-0.129	-0.111	-0.282	0.038	0.147	-0.102	0.010	**0.853**	**1**	**-0.573**	**-0.418**	-0.042	-0.003	0.028	0.012	0.051	0.051	0.051
Profile Curvature	0.100	0.024	-0.003	-0.145	-0.253	**-0.333**	-0.143	-0.030	-0.007	-0.034	0.064	0.094	0.270	0.001	0.083	-0.072	-0.100	-0.061	-0.038	-0.271	-0.271	-0.268	0.165	-0.128	-0.011	0.131	0.200	-0.179	0.010	0.076	**-0.917**	**-0.573**	**1**	0.169	0.014	-0.122	-0.081	-0.133	-0.001	-0.002	-0.002
TWI	0.050	-0.157	-0.007	0.171	0.275	0.107	-0.283	-0.218	0.003	-0.275	-0.027	-0.118	0.076	0.229	0.235	-0.231	-0.102	-0.113	-0.187	-0.037	-0.038	-0.036	0.153	-0.121	-0.016	-0.152	0.140	**-0.459**	0.018	-0.038	-0.311	**-0.418**	0.169	**1**	-0.021	**-0.325**	-0.268	-0.323	0.001	0.000	0.000
DEM	**-0.367**	0.178	**0.968**	0.000	-0.118	0.093	-0.190	-0.164	-0.091	-0.162	-0.279	-0.205	-0.269	-0.155	0.123	0.014	0.323	0.168	0.163	0.188	0.188	0.187	-0.145	0.243	**0.330**	-0.069	0.153	0.271	-0.095	-0.114	-0.029	-0.042	0.014	-0.021	**1**	**-0.421**	**-0.507**	**-0.447**	-0.135	-0.136	-0.136

BIO01	0.226	-0.132	**-0.401**	**0.388**	-0.162	-0.217	0.102	**0.484**	0.113	**0.549**	0.125	0.211	**-0.451**	**-0.424**	**-0.804**	**0.646**	-0.157	0.072	0.056	-0.150	-0.150	-0.154	0.165	**0.489**	-0.098	**0.423**	**-0.641**	0.083	0.273	**0.381**	0.076	-0.003	-0.122	**-0.325**	**-0.421**	**1**	**0.867**	**0.983**	-0.121	-0.119	-0.119
BIO05	0.232	-0.131	**-0.483**	**0.383**	-0.016	-0.224	0.128	**0.387**	0.078	**0.445**	0.165	0.219	**-0.358**	**-0.459**	**-0.672**	**0.415**	-0.176	-0.022	-0.126	-0.135	-0.135	-0.136	0.132	**0.408**	**-0.350**	0.279	**-0.522**	-0.058	0.170	0.244	0.065	0.028	-0.081	-0.268	**-0.507**	**0.867**	**1**	**0.888**	0.179	0.181	0.181
BIO06	0.216	-0.185	**-0.430**	**0.410**	-0.167	-0.225	0.135	**0.552**	0.127	**0.628**	0.155	0.231	**-0.484**	**-0.456**	**-0.859**	**0.660**	-0.180	0.144	0.092	-0.148	-0.148	-0.151	0.122	**0.431**	-0.113	**0.460**	**-0.694**	0.055	0.215	**0.355**	0.090	0.012	-0.133	-0.323	**-0.447**	**0.983**	**0.888**	**1**	-0.083	-0.080	-0.081
BIO12	**-0.343**	0.265	-0.193	-0.192	0.127	-0.024	0.060	0.073	0.092	0.043	0.160	0.094	0.146	-0.133	**0.332**	**-0.434**	-0.111	-0.087	0.022	**0.422**	**0.422**	**0.427**	**-0.361**	**-0.409**	**-0.524**	-0.144	0.194	-0.185	-0.191	-0.264	0.025	0.051	-0.001	0.001	-0.135	-0.121	0.179	-0.083	**1**	**1.000**	**1.000**
BIO13	**-0.343**	0.265	-0.193	-0.191	0.126	-0.025	0.061	0.075	0.093	0.045	0.160	0.094	0.145	-0.134	**0.330**	**-0.432**	-0.111	-0.086	0.023	**0.422**	**0.422**	**0.427**	**-0.362**	**-0.408**	**-0.523**	-0.143	0.192	-0.185	-0.191	-0.263	0.026	0.051	-0.002	0.000	-0.136	-0.119	0.181	-0.080	**1.000**	**1**	**1.000**
BIO14	**-0.343**	0.265	-0.194	-0.191	0.126	-0.025	0.060	0.075	0.093	0.045	0.160	0.094	0.145	-0.134	**0.330**	**-0.433**	-0.111	-0.086	0.023	**0.422**	**0.422**	**0.427**	**-0.362**	**-0.408**	**-0.524**	-0.143	0.192	-0.185	-0.191	-0.263	0.026	0.051	-0.002	0.000	-0.136	-0.119	0.181	-0.081	**1.000**	**1.000**	**1**

p-values (Pearson):

Variables	IC50 Average (mg/ml)	DPPH activity IC50 (μg/ml)	Elevation (m)	Soil Bearing Capacity (ts/f)	SM150T output (V)	pH	Electrical Conductivity (EC) (mS/cm)	Nitrogen (mg/100 g)	Ammonium-nitrogen (NH4+) (mg/100 g)	Nitrate – nitrogen (NO3) (mg/100 g)	Available Phosphorus (P) (mg/100 g)	Exchangeable Potassium (mg/100 g)	Exchangeable Calcium (Ca2+) (mg/100 g)	Exchangeable Magnesium (mg/100 g)	Silt (%)	Sand (%)	pH in H2O	Organic Carbon Density (kg/m3)	Organic Carbon Content (g/Kg)	Clay (%)	Cation Exchange Capacity (mmol(c)/kg)	Bulk Density (Kg/m3)	Distance to Road (m)	Distance to Stream (m)	Distance to Urban (m)	Annual Mean Temperature (° C)	Annual Mean Precipitation (mm)	Slope (°)	Aspect (°)	Hillshade (°)	Curvature	Plan Curvature	Profile Curvature	TWI	DEM	BIO01	BIO05	BIO06	BIO12	BIO13	BIO14
IC50 Average (mg/ml)	**0**	0.230	0.091	0.118	0.937	0.867	0.395	0.686	0.659	0.769	0.906	0.659	0.672	0.586	0.331	0.544	0.219	0.858	0.282	**0.025**	**0.025**	**0.025**	**0.030**	0.619	0.546	0.750	0.188	0.294	0.408	0.405	0.640	0.851	0.554	0.767	**0.026**	0.179	0.166	0.200	**0.038**	**0.038**	**0.038**
DPPH activity IC50 (μg/ml)	0.230	**0**	0.561	0.212	0.212	0.476	0.490	0.915	0.792	0.999	0.816	0.233	0.104	0.367	0.073	0.704	0.672	0.439	0.361	0.096	0.096	0.094	0.844	0.554	0.287	0.794	0.214	0.144	0.999	0.492	0.951	0.758	0.889	0.353	0.293	0.434	0.440	0.272	0.112	0.113	0.113
Elevation (m)	0.091	0.561	**0**	0.734	0.608	0.222	0.333	0.272	0.667	0.239	0.092	0.202	0.091	0.401	0.547	0.741	**0.026**	0.265	0.427	0.865	0.863	0.869	0.608	0.066	0.071	0.708	0.577	0.167	0.738	0.659	0.988	0.997	0.984	0.966	**<0.0001**	**0.014**	**0.002**	**0.008**	0.253	0.252	0.251
Soil Bearing Capacity (ts/f)	0.118	0.212	0.734	**0**	0.136	0.263	0.984	0.812	0.578	0.565	**0.028**	0.226	**0.000**	0.095	**0.002**	0.145	0.310	0.802	0.125	0.426	0.423	0.415	0.099	**0.021**	0.933	0.703	**<0.0001**	0.117	**0.042**	0.181	0.844	0.478	0.393	0.313	1.000	**0.018**	**0.019**	**0.012**	0.256	0.258	0.258
SM150T output (V)	0.937	0.212	0.608	0.136	**0**	0.508	0.096	**<0.0001**	**0.005**	**0.000**	0.414	0.162	0.969	0.334	0.122	**0.006**	0.431	**0.006**	**<0.0001**	0.866	0.874	0.866	0.575	0.789	**0.026**	**0.021**	0.257	**0.036**	0.301	0.449	0.207	0.542	0.130	0.100	0.485	0.337	0.927	0.323	0.453	0.456	0.456
pH	0.867	0.476	0.222	0.263	0.508	**0**	0.278	0.656	0.070	0.723	0.919	**0.041**	0.233	0.735	0.278	0.778	0.392	0.553	0.493	0.362	0.366	0.362	0.944	0.460	0.883	0.121	0.766	0.754	0.729	0.858	**0.035**	0.093	**0.044**	0.529	0.585	0.197	0.183	0.180	0.887	0.885	0.885

Electrical Conductivity (EC) (mS/cm)	0.395	0.490	0.333	0.984	0.096	0.278	**0**	0.251	0.797	0.187	0.280	0.092	0.926	0.237	0.716	0.637	0.515	**0.029**	0.296	0.959	0.954	0.965	0.564	0.990	0.224	0.193	0.171	0.590	**0.001**	**0.006**	0.540	0.870	0.399	0.090	0.261	0.548	0.449	0.427	0.724	0.722	0.722
Nitrogen (mg/100g)	0.686	0.915	0.272	0.812	**<0.0001**	0.656	0.251	**0**	**<0.0001**	**<0.0001**	0.302	0.254	0.055	**0.005**	**0.000**	**<0.0001**	0.568	**0.002**	**<0.0001**	0.446	0.440	0.451	0.303	0.691	0.385	**0.001**	**<0.0001**	0.075	0.491	0.253	0.954	0.733	0.860	0.195	0.332	**0.002**	**0.018**	**0.000**	0.668	0.660	0.661
Ammonium-nitrogen (NH_4^+) (mg/100 g)	0.659	0.792	0.667	0.578	**0.005**	0.070	0.797	**<0.0001**	**0**	0.058	0.291	0.703	0.731	0.282	0.311	0.201	0.317	0.170	**0.003**	0.915	0.907	0.910	0.354	0.218	0.832	0.597	0.099	0.111	0.814	0.055	0.949	0.864	0.969	0.986	0.591	0.505	0.646	0.454	0.588	0.585	0.585
Nitrate – nitrogen (NO_3) (mg/100 g)	0.769	0.999	0.239	0.565	**0.000**	0.723	0.187	**<0.0001**	0.058	**0**	0.449	0.209	**0.024**	**0.003**	**<0.0001**	**<0.0001**	0.838	**0.001**	**0.000**	0.365	0.361	0.373	0.419	0.257	0.321	**<0.0001**	**<0.0001**	0.159	0.452	0.648	0.965	0.730	0.842	0.100	0.337	**0.000**	**0.006**	**<0.0001**	0.800	0.791	0.792
Available Phosphorus (P) (mg/100 g)	0.906	0.816	0.092	**0.028**	0.414	0.919	0.280	0.302	0.291	0.449	**0**	**0.012**	0.310	0.174	0.591	0.568	0.052	0.338	0.190	0.880	0.882	0.883	0.232	0.137	0.352	0.694	0.525	0.532	0.792	**0.031**	0.890	0.836	0.708	0.876	0.094	0.461	0.330	0.359	0.345	0.344	0.344
Exchangeable Potassium (mg/100 g)	0.659	0.233	0.202	0.226	0.162	**0.041**	0.092	0.254	0.703	0.209	**0.012**	**0**	0.234	0.704	0.241	0.175	0.281	**0.024**	0.053	0.497	0.495	0.497	0.797	0.464	0.886	**0.000**	0.509	0.828	**0.022**	0.747	0.525	0.561	0.582	0.485	0.223	0.211	0.192	0.170	0.582	0.579	0.579
Exchangeable Calcium (Ca^{2+}) (mg/100 g)	0.672	0.104	0.091	**0.000**	0.969	0.233	0.926	0.055	0.731	**0.024**	0.310	0.234	**0**	**<0.0001**	**<0.0001**	**0.003**	0.374	0.346	0.687	0.338	0.338	0.348	0.241	**0.001**	0.846	**0.005**	**0.000**	0.803	0.647	0.350	0.358	0.841	0.106	0.657	0.107	**0.005**	**0.030**	**0.002**	0.387	0.391	0.391
Exchangeable Magnesium (mg/100 g)	0.586	0.367	0.401	0.095	0.334	0.735	0.237	**0.005**	0.282	**0.003**	0.174	0.704	**<0.0001**	**0**	**0.003**	**0.047**	0.141	0.321	0.960	0.878	0.879	0.876	0.241	**0.029**	**0.005**	0.094	**0.023**	0.960	0.508	0.361	0.836	0.670	0.997	0.172	0.360	**0.009**	**0.004**	**0.005**	0.431	0.427	0.428
Silt (%)	0.331	0.073	0.547	**0.002**	0.122	0.278	0.716	**0.000**	0.311	**<0.0001**	0.591	0.241	**<0.0001**	**0.003**	**0**	**<0.0001**	0.415	**0.044**	0.288	0.164	0.164	0.155	0.432	**0.002**	0.508	**0.001**	**<0.0001**	0.529	0.351	**0.011**	0.911	0.683	0.624	0.162	0.467	**<0.0001**	**<0.0001**	**<0.0001**	**0.045**	**0.046**	**0.046**
Sand (%)	0.544	0.704	0.741	0.145	**0.006**	0.778	0.637	**<0.0001**	0.201	**<0.0001**	0.568	0.175	**0.003**	**0.047**	**<0.0001**	**0**	0.584	**0.000**	**0.000**	0.124	0.126	0.117	0.269	**<0.0001**	**0.039**	**<0.0001**	**<0.0001**	**0.013**	0.924	0.250	0.914	0.438	0.671	0.168	0.933	**<0.0001**	**0.011**	**<0.0001**	**0.007**	**0.008**	**0.008**
pH in H_2O	0.219	0.672	**0.026**	0.310	0.431	0.392	0.515	0.568	0.317	0.838	0.052	0.281	0.374	0.141	0.415	0.584	**0**	0.411	0.620	0.668	0.670	0.668	0.058	**0.017**	0.479	0.426	0.529	0.056	0.523	**0.004**	0.449	0.435	0.557	0.549	0.051	0.354	0.297	0.287	0.513	0.512	0.512
Organic Carbon Density (kg/m3)	0.858	0.439	0.265	0.802	**0.006**	0.553	**0.029**	**0.002**	0.170	**0.001**	0.338	**0.024**	0.346	0.321	**0.044**	**0.000**	0.411	**0**	**<0.0001**	0.457	0.463	0.449	0.728	0.535	0.180	**0.018**	**0.014**	0.051	**0.007**	0.322	0.952	0.555	0.720	0.507	0.320	0.670	0.895	0.397	0.609	0.613	0.612
Organic Carbon Content (g/Kg)	0.282	0.361	0.427	0.125	**<0.0001**	0.493	0.296	**<0.0001**	**0.003**	**0.000**	0.190	0.053	0.687	0.960	0.288	**0.000**	0.620	**<0.0001**	**0**	0.288	0.282	0.292	0.335	0.686	**0.012**	**0.028**	**0.029**	**0.001**	**0.048**	0.930	0.988	0.796	0.822	0.268	0.335	0.741	0.456	0.588	0.896	0.892	0.892
Clay (%)	**0.025**	0.096	0.865	0.426	0.866	0.362	0.959	0.446	0.915	0.365	0.880	0.497	0.338	0.878	0.164	0.124	0.668	0.457	0.288	**0**	**<0.0001**	**<0.0001**	**0.008**	0.329	0.367	0.713	0.850	0.466	0.476	0.490	0.286	0.927	0.105	0.827	0.265	0.374	0.427	0.383	**0.009**	**0.009**	**0.009**

Cation Exchange Capacity (mmol(c)/kg)	**0.025**	0.096	0.863	0.423	0.874	0.366	0.954	0.440	0.907	0.361	0.882	0.495	0.338	0.879	0.164	0.126	0.670	0.463	0.282	**<0.0001**	**0**	**<0.0001**	**0.008**	0.330	0.364	0.710	0.854	0.460	0.473	0.490	0.285	0.927	0.105	0.825	0.264	0.374	0.426	0.383	**0.009**	**0.009**	**0.009**
Bulk Density (Kg/m3)	**0.025**	0.094	0.869	0.415	0.866	0.362	0.965	0.451	0.910	0.373	0.883	0.497	0.348	0.876	0.155	0.117	0.668	0.449	0.292	**<0.0001**	**<0.0001**	**0**	**0.007**	0.318	0.380	0.729	0.829	0.476	0.470	0.482	0.290	0.927	0.109	0.834	0.267	0.362	0.423	0.371	**0.008**	**0.008**	**0.008**
Distance to Road (m)	**0.030**	0.844	0.608	0.099	0.575	0.944	0.564	0.303	0.354	0.419	0.232	0.797	0.241	0.241	0.432	0.269	0.058	0.728	0.335	**0.008**	**0.008**	**0.007**	**0**	0.121	0.092	0.859	0.889	0.440	0.136	0.208	0.167	0.119	0.330	0.365	0.393	0.330	0.437	0.472	**0.028**	**0.028**	**0.028**
Distance to Stream (m)	0.619	0.554	0.066	**0.021**	0.789	0.460	0.990	0.691	0.218	0.257	0.137	0.464	**0.001**	**0.029**	**0.002**	**<0.0001**	**0.017**	0.535	0.686	0.329	0.330	0.318	0.121	**0**	0.371	**0.012**	**0.015**	0.147	0.341	0.983	0.914	0.447	0.452	0.476	0.147	**0.002**	**0.012**	**0.008**	**0.012**	**0.012**	**0.012**
Distance to Urban (m)	0.546	0.287	0.071	0.933	**0.026**	0.883	0.224	0.385	0.832	0.321	0.352	0.886	0.846	**0.005**	0.508	**0.039**	0.479	0.180	**0.012**	0.367	0.364	0.380	0.092	0.371	**0**	0.385	0.368	0.053	0.423	0.060	0.783	0.514	0.947	0.927	**0.046**	0.564	**0.034**	0.504	**0.001**	**0.001**	**0.001**
Annual Mean Temperature (° C)	0.750	0.794	0.708	0.703	**0.021**	0.121	0.193	**0.001**	0.597	**<0.0001**	0.694	**0.000**	**0.005**	0.094	**0.001**	**<0.0001**	0.426	**0.018**	**0.028**	0.713	0.710	0.729	0.859	**0.012**	0.385	**0**	**0.002**	0.359	0.631	0.848	0.189	0.091	0.440	0.370	0.685	**0.009**	0.094	**0.004**	0.395	0.399	0.399
Annual Mean Precipitation (mm)	0.188	0.214	0.577	**<0.0001**	0.257	0.766	0.171	**<0.0001**	0.099	**<0.0001**	0.525	0.509	**0.000**	**0.023**	**<0.0001**	**<0.0001**	0.529	**0.014**	**0.029**	0.850	0.854	0.829	0.889	**0.015**	0.368	**0.002**	**0**	0.392	0.432	0.081	0.521	0.824	0.235	0.410	0.365	**<0.0001**	**0.001**	**<0.0001**	0.251	0.255	0.255
Slope (°)	0.294	0.144	0.167	0.117	**0.036**	0.754	0.590	0.075	0.111	0.159	0.532	0.828	0.803	0.960	0.529	**0.013**	0.056	0.051	**0.001**	0.466	0.460	0.476	0.440	0.147	0.053	0.359	0.392	**0**	0.725	0.854	0.272	0.385	0.290	**0.004**	0.105	0.624	0.734	0.749	0.272	0.274	0.274
Aspect (°)	0.408	0.999	0.738	**0.042**	0.301	0.729	**0.001**	0.491	0.814	0.452	0.792	**0.022**	0.647	0.508	0.351	0.924	0.523	**0.007**	**0.048**	0.476	0.473	0.470	0.136	0.341	0.423	0.631	0.432	0.725	**0**	**<0.0001**	0.744	0.547	0.955	0.915	0.577	0.102	0.314	0.201	0.256	0.257	0.257
Hillshade (°)	0.405	0.492	0.659	0.181	0.449	0.858	**0.006**	0.253	0.055	0.648	**0.031**	0.747	0.350	0.361	**0.011**	0.250	**0.004**	0.322	0.930	0.490	0.490	0.482	0.208	0.983	0.060	0.848	0.081	0.854	**<0.0001**	**0**	0.797	0.953	0.653	0.824	0.502	**0.020**	0.146	**0.031**	0.115	0.116	0.116
Curvature	0.640	0.951	0.988	0.844	0.207	**0.035**	0.540	0.954	0.949	0.965	0.890	0.525	0.358	0.836	0.911	0.914	0.449	0.952	0.988	0.286	0.285	0.290	0.167	0.914	0.783	0.189	0.521	0.272	0.744	0.797	**0**	**<0.0001**	**<0.0001**	0.061	0.865	0.655	0.704	0.595	0.881	0.880	0.880
Plan Curvature	0.851	0.758	0.997	0.478	0.542	0.093	0.870	0.733	0.864	0.730	0.836	0.561	0.841	0.670	0.683	0.438	0.435	0.555	0.796	0.927	0.927	0.927	0.119	0.447	0.514	0.091	0.824	0.385	0.547	0.953	**<0.0001**	**0**	**0.000**	**0.010**	0.807	0.985	0.870	0.943	0.765	0.765	0.764
Profile Curvature	0.554	0.889	0.984	0.393	0.130	**0.044**	0.399	0.860	0.969	0.842	0.708	0.582	0.106	0.997	0.624	0.671	0.557	0.720	0.822	0.105	0.105	0.109	0.330	0.452	0.947	0.440	0.235	0.290	0.955	0.653	**<0.0001**	**0.000**	**0**	0.317	0.936	0.473	0.636	0.433	0.995	0.993	0.993
TWI	0.767	0.353	0.966	0.313	0.100	0.529	0.090	0.195	0.986	0.100	0.876	0.485	0.657	0.172	0.162	0.168	0.549	0.507	0.268	0.827	0.825	0.834	0.365	0.476	0.927	0.370	0.410	**0.004**	0.915	0.824	0.061	**0.010**	0.317	**0**	0.902	**0.050**	0.109	0.052	0.994	0.999	0.998
DEM	**0.026**	0.293	**<0.0001**	1.000	0.485	0.585	0.261	0.332	0.591	0.337	0.094	0.223	0.107	0.360	0.467	0.933	0.051	0.320	0.335	0.265	0.264	0.267	0.393	0.147	**0.046**	0.685	0.365	0.105	0.577	0.502	0.865	0.807	0.936	0.902	**0**	**0.010**	**0.001**	**0.006**	0.426	0.423	0.423
BIO01	0.179	0.434	**0.014**	**0.018**	0.337	0.197	0.548	**0.002**	0.505	**0.000**	0.461	0.211	**0.005**	**0.009**	**<0.0001**	**<0.0001**	0.354	0.670	0.741	0.374	0.374	0.362	0.330	**0.002**	0.564	**0.009**	**<0.0001**	0.624	0.102	**0.020**	0.655	0.985	0.473	**0.050**	**0.010**	**0**	**<0.0001**	**<0.0001**	0.474	0.482	0.482
BIO05	0.166	0.440	**0.002**	**0.019**	0.927	0.183	0.449	**0.018**	0.646	**0.006**	0.330	0.192	**0.030**	**0.004**	**<0.0001**	**0.011**	0.297	0.895	0.456	0.427	0.426	0.423	0.437	**0.012**	**0.034**	0.094	**0.001**	0.734	0.314	0.146	0.704	0.870	0.636	0.109	**0.001**	**<0.0001**	**0**	**<0.0001**	0.290	0.285	0.285
BIO06	0.200	0.272	**0.008**	**0.012**	0.323	0.180	0.427	**0.000**	0.454	**<0.0001**	0.359	0.170	**0.002**	**0.005**	**<0.0001**	**<0.0001**	0.287	0.397	0.588	0.383	0.383	0.371	0.472	**0.008**	0.504	**0.004**	**<0.0001**	0.749	0.201	**0.031**	0.595	0.943	0.433	0.052	**0.006**	**<0.0001**	**<0.0001**	**0**	0.626	0.636	0.636
BIO12	**0.038**	0.112	0.253	0.256	0.453	0.887	0.724	0.668	0.588	0.800	0.345	0.582	0.387	0.431	**0.045**	**0.007**	0.513	0.609	0.896	**0.009**	**0.009**	**0.008**	**0.028**	**0.012**	**0.001**	0.395	0.251	0.272	0.256	0.115	0.881	0.765	0.995	0.994	0.426	0.474	0.290	0.626	**0**	**<0.0001**	**<0.0001**
BIO13	**0.038**	0.113	0.252	0.258	0.456	0.885	0.722	0.660	0.585	0.791	0.344	0.579	0.391	0.427	**0.046**	**0.008**	0.512	0.613	0.892	**0.009**	**0.009**	**0.008**	**0.028**	**0.012**	**0.001**	0.399	0.255	0.274	0.257	0.116	0.880	0.765	0.993	0.999	0.423	0.482	0.285	0.636	**<0.0001**	**0**	**<0.0001**

BIO14	**0.038**	0.113	0.251	0.258	0.456	0.885	0.722	0.661	0.585	0.792	0.344	0.579	0.391	0.428	**0.046**	**0.008**	0.512	0.612	0.892	**0.009**	**0.009**	**0.008**	**0.028**	**0.012**	**0.001**	0.399	0.255	0.274	0.257	0.116	0.880	0.764	0.993	0.998	0.423	0.482	0.285	0.636	**<0.0001**	**<0.0001**	**0**

Coefficients of determination (Pearson):

Variables	IC50 Average (mg/ml)	DPPH activity IC50 (µg/ml)	Elevation (m)	Soil Bearing Capacity (ts/f)	SM150T output (V)	pH	Electrical Conductivity (EC) (mS/cm)	Nitrogen (mg/100g)	Ammonium-nitrogen (NH4+) (mg/100 g)	Nitrate – nitrogen (NO3) (mg/100 g)	Available Phosphorus (P) (mg/100 g)	Exchangeable Potassium (mg/100 g)	Exchangeable Calcium (Ca2+) (mg/100 g)	Exchangeable Magnesium (mg/100 g)	Silt (%)	Sand (%)	pH in H2O	Organic Carbon Density (kg/m3)	Organic Carbon Content (g/Kg)	Clay (%)	Cation Exchange Capacity (mmol(c)/kg)	Bulk Density (Kg/m3)	Distance to Road (m)	Distance to Stream (m)	Distance to Urban (m)	Annual Mean Temperature (° C)	Annual Mean Precipitation (mm)	Slope (°)	Aspect (°)	Hillshade (°)	Curvature	Plan Curvature	Profile Curvature	TWI	DEM	BIO01	BIO05	BIO06	BIO12	BIO13	BIO14
IC50 Average (mg/ml)	**1**	0.041	0.080	0.068	0.000	0.001	0.021	0.005	0.006	0.002	0.000	0.006	0.005	0.009	0.027	0.011	0.043	0.001	0.033	0.135	0.135	0.135	0.128	0.007	0.011	0.003	0.049	0.031	0.020	0.020	0.006	0.001	0.010	0.003	0.135	0.051	0.054	0.047	0.118	0.118	0.118
DPPH activity IC50 (µg/ml)	0.041	**1**	0.010	0.044	0.044	0.015	0.014	0.000	0.002	0.000	0.002	0.040	0.074	0.023	0.089	0.004	0.005	0.017	0.024	0.077	0.077	0.078	0.001	0.010	0.032	0.002	0.044	0.060	0.000	0.014	0.000	0.003	0.001	0.025	0.032	0.018	0.017	0.034	0.070	0.070	0.070
Elevation (m)	0.080	0.010	**1**	0.003	0.008	0.042	0.027	0.034	0.005	0.039	0.079	0.046	0.079	0.020	0.010	0.003	0.134	0.035	0.018	0.001	0.001	0.001	0.008	0.093	0.090	0.004	0.009	0.054	0.003	0.006	0.000	0.000	0.000	0.000	0.938	0.161	0.234	0.185	0.037	0.037	0.037
Soil Bearing Capacity (ts/f)	0.068	0.044	0.003	**1**	0.062	0.036	0.000	0.002	0.009	0.010	0.131	0.042	0.318	0.078	0.241	0.060	0.029	0.002	0.066	0.018	0.018	0.019	0.076	0.143	0.000	0.004	0.358	0.069	0.113	0.051	0.001	0.014	0.021	0.029	0.000	0.150	0.146	0.168	0.037	0.036	0.036
SM150T output (V)	0.000	0.044	0.008	0.062	**1**	0.013	0.077	0.385	0.208	0.298	0.019	0.055	0.000	0.027	0.067	0.194	0.018	0.196	0.386	0.001	0.001	0.001	0.009	0.002	0.134	0.144	0.037	0.120	0.031	0.016	0.045	0.011	0.064	0.076	0.014	0.026	0.000	0.028	0.016	0.016	0.016
pH	0.001	0.015	0.042	0.036	0.013	**1**	0.034	0.006	0.091	0.004	0.000	0.114	0.040	0.003	0.034	0.002	0.021	0.010	0.014	0.024	0.023	0.024	0.000	0.016	0.001	0.067	0.003	0.003	0.003	0.001	0.121	0.078	0.111	0.011	0.009	0.047	0.050	0.051	0.001	0.001	0.001
Electrical Conductivity (EC) (mS/cm)	0.021	0.014	0.027	0.000	0.077	0.034	**1**	0.038	0.002	0.049	0.033	0.079	0.000	0.040	0.004	0.006	0.012	0.129	0.031	0.000	0.000	0.000	0.010	0.000	0.042	0.048	0.053	0.008	0.272	0.195	0.011	0.001	0.020	0.080	0.036	0.010	0.016	0.018	0.004	0.004	0.004
Nitrogen (mg/100g)	0.005	0.000	0.034	0.002	0.385	0.006	0.038	**1**	0.434	0.848	0.030	0.037	0.101	0.204	0.339	0.379	0.009	0.244	0.437	0.017	0.017	0.016	0.030	0.005	0.022	0.279	0.457	0.088	0.014	0.037	0.000	0.003	0.001	0.048	0.027	0.234	0.150	0.304	0.005	0.006	0.006
Ammonium-nitrogen (NH4+) (mg/100 g)	0.006	0.002	0.005	0.009	0.208	0.091	0.002	0.434	**1**	0.099	0.032	0.004	0.003	0.033	0.029	0.046	0.029	0.053	0.219	0.000	0.000	0.000	0.025	0.043	0.001	0.008	0.076	0.071	0.002	0.102	0.000	0.001	0.000	0.000	0.008	0.013	0.006	0.016	0.008	0.009	0.009
Nitrate – nitrogen (NO3) (mg/100 g)	0.002	0.000	0.039	0.010	0.298	0.004	0.049	0.848	0.099	**1**	0.016	0.045	0.137	0.224	0.413	0.443	0.001	0.257	0.352	0.023	0.024	0.023	0.019	0.037	0.028	0.386	0.505	0.056	0.016	0.006	0.000	0.003	0.001	0.075	0.026	0.302	0.198	0.394	0.002	0.002	0.002
Available Phosphorus (P) (mg/100 g)	0.000	0.002	0.079	0.131	0.019	0.000	0.033	0.030	0.032	0.016	**1**	0.166	0.029	0.052	0.008	0.009	0.103	0.026	0.049	0.001	0.001	0.001	0.040	0.062	0.025	0.004	0.012	0.011	0.002	0.125	0.001	0.001	0.004	0.001	0.078	0.016	0.027	0.024	0.025	0.026	0.026

Exchangeable Potassium (mg/100 g)	0.006	0.040	0.046	0.042	0.055	0.114	0.079	0.037	0.004	0.045	0.166	**1**	0.040	0.004	0.039	0.052	0.033	0.137	0.103	0.013	0.013	0.013	0.002	0.015	0.001	0.311	0.013	0.001	0.141	0.003	0.012	0.010	0.009	0.014	0.042	0.044	0.048	0.053	0.009	0.009	0.009
Exchangeable Calcium (Ca2+) (mg/100 g)	0.005	0.074	0.079	0.318	0.000	0.040	0.000	0.101	0.003	0.137	0.029	0.040	**1**	0.372	0.407	0.222	0.023	0.025	0.005	0.026	0.026	0.025	0.039	0.274	0.001	0.207	0.339	0.002	0.006	0.025	0.024	0.001	0.073	0.006	0.072	0.204	0.128	0.234	0.021	0.021	0.021
Exchangeable Magnesium (mg/100 g)	0.009	0.023	0.020	0.078	0.027	0.003	0.040	0.204	0.033	0.224	0.052	0.004	0.372	**1**	0.221	0.108	0.061	0.028	0.000	0.001	0.001	0.001	0.039	0.129	0.201	0.078	0.138	0.000	0.013	0.024	0.001	0.005	0.000	0.053	0.024	0.179	0.210	0.208	0.018	0.018	0.018
Silt (%)	0.027	0.089	0.010	0.241	0.067	0.034	0.004	0.339	0.029	0.413	0.008	0.039	0.407	0.221	**1**	0.603	0.019	0.111	0.032	0.055	0.054	0.057	0.018	0.239	0.013	0.294	0.559	0.011	0.025	0.171	0.000	0.005	0.007	0.055	0.015	0.646	0.452	0.737	0.110	0.109	0.109
Sand (%)	0.011	0.004	0.003	0.060	0.194	0.002	0.006	0.379	0.046	0.443	0.009	0.052	0.222	0.108	0.603	**1**	0.009	0.326	0.310	0.066	0.065	0.069	0.035	0.363	0.116	0.358	0.477	0.163	0.000	0.038	0.000	0.017	0.005	0.054	0.000	0.418	0.172	0.435	0.189	0.187	0.187
pH in H2O	0.043	0.005	0.134	0.029	0.018	0.021	0.012	0.009	0.029	0.001	0.103	0.033	0.023	0.061	0.019	0.009	**1**	0.019	0.007	0.005	0.005	0.005	0.099	0.153	0.014	0.018	0.011	0.101	0.012	0.216	0.016	0.018	0.010	0.010	0.104	0.025	0.031	0.032	0.012	0.012	0.012
Organic Carbon Density (kg/m3)	0.001	0.017	0.035	0.002	0.196	0.010	0.129	0.244	0.053	0.257	0.026	0.137	0.025	0.028	0.111	0.326	0.019	**1**	0.601	0.016	0.015	0.016	0.004	0.011	0.051	0.149	0.160	0.105	0.192	0.028	0.000	0.010	0.004	0.013	0.028	0.005	0.001	0.021	0.008	0.007	0.007
Organic Carbon Content (g/Kg)	0.033	0.024	0.018	0.066	0.386	0.014	0.031	0.437	0.219	0.352	0.049	0.103	0.005	0.000	0.032	0.310	0.007	0.601	**1**	0.032	0.033	0.032	0.027	0.005	0.167	0.131	0.129	0.263	0.107	0.000	0.000	0.002	0.001	0.035	0.027	0.003	0.016	0.008	0.000	0.001	0.001
Clay (%)	0.135	0.077	0.001	0.018	0.001	0.024	0.000	0.017	0.000	0.023	0.001	0.013	0.026	0.001	0.055	0.066	0.005	0.016	0.032	**1**	1.000	1.000	0.185	0.027	0.023	0.004	0.001	0.015	0.015	0.014	0.032	0.000	0.073	0.001	0.035	0.023	0.018	0.022	0.178	0.178	0.178
Cation Exchange Capacity (mmol(c)/kg)	0.135	0.077	0.001	0.018	0.001	0.023	0.000	0.017	0.000	0.024	0.001	0.013	0.026	0.001	0.054	0.065	0.005	0.015	0.033	1.000	**1**	1.000	0.185	0.027	0.024	0.004	0.001	0.016	0.015	0.014	0.033	0.000	0.073	0.001	0.036	0.023	0.018	0.022	0.178	0.178	0.178
Bulk Density (Kg/m3)	0.135	0.078	0.001	0.019	0.001	0.024	0.000	0.016	0.000	0.023	0.001	0.013	0.025	0.001	0.057	0.069	0.005	0.016	0.032	1.000	1.000	**1**	0.188	0.029	0.022	0.003	0.001	0.015	0.015	0.014	0.032	0.000	0.072	0.001	0.035	0.024	0.018	0.023	0.183	0.183	0.183
Distance to Road (m)	0.128	0.001	0.008	0.076	0.009	0.000	0.010	0.030	0.025	0.019	0.040	0.002	0.039	0.039	0.018	0.035	0.099	0.004	0.027	0.185	0.185	0.188	**1**	0.067	0.079	0.001	0.001	0.017	0.062	0.045	0.054	0.068	0.027	0.024	0.021	0.027	0.017	0.015	0.131	0.131	0.131
Distance to Stream (m)	0.007	0.010	0.093	0.143	0.002	0.016	0.000	0.005	0.043	0.037	0.062	0.015	0.274	0.129	0.239	0.363	0.153	0.011	0.005	0.027	0.027	0.029	0.067	**1**	0.023	0.167	0.158	0.059	0.026	0.000	0.000	0.017	0.016	0.015	0.059	0.239	0.167	0.185	0.167	0.166	0.167
Distance to Urban (m)	0.011	0.032	0.090	0.000	0.134	0.001	0.042	0.022	0.001	0.028	0.025	0.001	0.001	0.201	0.013	0.116	0.014	0.051	0.167	0.023	0.024	0.022	0.079	0.023	**1**	0.022	0.023	0.103	0.018	0.097	0.002	0.012	0.000	0.000	0.109	0.010	0.123	0.013	0.274	0.274	0.274
Annual Mean Temperature (° C)	0.003	0.002	0.004	0.004	0.144	0.067	0.048	0.279	0.008	0.386	0.004	0.311	0.207	0.078	0.294	0.358	0.018	0.149	0.131	0.004	0.004	0.003	0.001	0.167	0.022	**1**	0.239	0.024	0.007	0.001	0.049	0.079	0.017	0.023	0.005	0.179	0.078	0.211	0.021	0.020	0.020
Annual Mean	0.049	0.044	0.009	0.358	0.037	0.003	0.053	0.457	0.076	0.505	0.012	0.013	0.339	0.138	0.559	0.477	0.011	0.160	0.129	0.001	0.001	0.001	0.001	0.158	0.023	0.239	**1**	0.021	0.018	0.085	0.012	0.001	0.040	0.020	0.023	0.411	0.272	0.481	0.038	0.037	0.037

Precipitation (mm)																																									
Slope (°)	0.031	0.060	0.054	0.069	0.120	0.003	0.008	0.088	0.071	0.056	0.011	0.001	0.002	0.000	0.011	0.163	0.101	0.105	0.263	0.015	0.016	0.015	0.017	0.059	0.103	0.024	0.021	**1**	0.004	0.001	0.034	0.022	0.032	0.211	0.073	0.007	0.003	0.003	0.034	0.034	0.034
Aspect (°)	0.020	0.000	0.003	0.113	0.031	0.003	0.272	0.014	0.002	0.016	0.002	0.141	0.006	0.013	0.025	0.000	0.012	0.192	0.107	0.015	0.015	0.015	0.062	0.026	0.018	0.007	0.018	0.004	**1**	0.469	0.003	0.010	0.000	0.000	0.009	0.075	0.029	0.046	0.037	0.037	0.037
Hillshade (°)	0.020	0.014	0.006	0.051	0.016	0.001	0.195	0.037	0.102	0.006	0.125	0.003	0.025	0.024	0.171	0.038	0.216	0.028	0.000	0.014	0.014	0.014	0.045	0.000	0.097	0.001	0.085	0.001	0.469	**1**	0.002	0.000	0.006	0.001	0.013	0.145	0.059	0.126	0.070	0.069	0.069
Curvature	0.006	0.000	0.000	0.001	0.045	0.121	0.011	0.000	0.000	0.000	0.001	0.012	0.024	0.001	0.000	0.000	0.016	0.000	0.000	0.032	0.033	0.032	0.054	0.000	0.002	0.049	0.012	0.034	0.003	0.002	**1**	0.727	0.840	0.097	0.001	0.006	0.004	0.008	0.001	0.001	0.001
Plan Curvature	0.001	0.003	0.000	0.014	0.011	0.078	0.001	0.003	0.001	0.003	0.001	0.010	0.001	0.005	0.005	0.017	0.018	0.010	0.002	0.000	0.000	0.000	0.068	0.017	0.012	0.079	0.001	0.022	0.010	0.000	0.727	**1**	0.328	0.175	0.002	0.000	0.001	0.000	0.003	0.003	0.003
Profile Curvature	0.010	0.001	0.000	0.021	0.064	0.111	0.020	0.001	0.000	0.001	0.004	0.009	0.073	0.000	0.007	0.005	0.010	0.004	0.001	0.073	0.073	0.072	0.027	0.016	0.000	0.017	0.040	0.032	0.000	0.006	0.840	0.328	**1**	0.029	0.000	0.015	0.006	0.018	0.000	0.000	0.000
TWI	0.003	0.025	0.000	0.029	0.076	0.011	0.080	0.048	0.000	0.075	0.001	0.014	0.006	0.053	0.055	0.054	0.010	0.013	0.035	0.001	0.001	0.001	0.024	0.015	0.000	0.023	0.020	0.211	0.000	0.001	0.097	0.175	0.029	**1**	0.000	0.106	0.072	0.104	0.000	0.000	0.000
DEM	0.135	0.032	0.938	0.000	0.014	0.009	0.036	0.027	0.008	0.026	0.078	0.042	0.072	0.024	0.015	0.000	0.104	0.028	0.027	0.035	0.036	0.035	0.021	0.059	0.109	0.005	0.023	0.073	0.009	0.013	0.001	0.002	0.000	0.000	**1**	0.177	0.257	0.199	0.018	0.018	0.018
BIO01	0.051	0.018	0.161	0.150	0.026	0.047	0.010	0.234	0.013	0.302	0.016	0.044	0.204	0.179	0.646	0.418	0.025	0.005	0.003	0.023	0.023	0.024	0.027	0.239	0.010	0.179	0.411	0.007	0.075	0.145	0.006	0.000	0.015	0.106	0.177	**1**	0.752	0.965	0.015	0.014	0.014
BIO05	0.054	0.017	0.234	0.146	0.000	0.050	0.016	0.150	0.006	0.198	0.027	0.048	0.128	0.210	0.452	0.172	0.031	0.001	0.016	0.018	0.018	0.018	0.017	0.167	0.123	0.078	0.272	0.003	0.029	0.059	0.004	0.001	0.006	0.072	0.257	0.752	**1**	0.788	0.032	0.033	0.033
BIO06	0.047	0.034	0.185	0.168	0.028	0.051	0.018	0.304	0.016	0.394	0.024	0.053	0.234	0.208	0.737	0.435	0.032	0.021	0.008	0.022	0.022	0.023	0.015	0.185	0.013	0.211	0.481	0.003	0.046	0.126	0.008	0.000	0.018	0.104	0.199	0.965	0.788	**1**	0.007	0.006	0.006
BIO12	0.118	0.070	0.037	0.037	0.016	0.001	0.004	0.005	0.008	0.002	0.025	0.009	0.021	0.018	0.110	0.189	0.012	0.008	0.000	0.178	0.178	0.183	0.131	0.167	0.274	0.021	0.038	0.034	0.037	0.070	0.001	0.003	0.000	0.000	0.018	0.015	0.032	0.007	**1**	1.000	1.000
BIO13	0.118	0.070	0.037	0.036	0.016	0.001	0.004	0.006	0.009	0.002	0.026	0.009	0.021	0.018	0.109	0.187	0.012	0.007	0.001	0.178	0.178	0.183	0.131	0.166	0.274	0.020	0.037	0.034	0.037	0.069	0.001	0.003	0.000	0.000	0.018	0.014	0.033	0.006	1.000	**1**	1.000
BIO14	0.118	0.070	0.037	0.036	0.016	0.001	0.004	0.006	0.009	0.002	0.026	0.009	0.021	0.018	0.109	0.187	0.012	0.007	0.001	0.178	0.178	0.183	0.131	0.167	0.274	0.020	0.037	0.034	0.037	0.069	0.001	0.003	0.000	0.000	0.018	0.014	0.033	0.006	1.000	1.000	**1**
